# TRIB3 promotes the progression of renal cell carcinoma by upregulating the lipid droplet-associated protein PLIN2

**DOI:** 10.1038/s41419-024-06627-4

**Published:** 2024-04-01

**Authors:** Jun Li, Qian Zhang, Yupeng Guan, Dingzhun Liao, Huikun Chen, Haiyun Xiong, Yiyu Sheng, Xianju Chen, Jun Pang

**Affiliations:** 1 Department of Urology, Kidney and Urology Center, The Seventh Affiliated Hospital, Sun Yat-sen University, Shenzhen, 518107 China; 2 Department of Rehabilitation Medicine, The Seventh Affiliated Hospital, Sun Yat-sen University, Shenzhen, 518107 China; 3 Department of Pathology, The Seventh Affiliated Hospital, Sun Yat-sen University, Shenzhen, 518107 China

**Keywords:** Urological cancer, Biomarkers

## Abstract

Abnormal lipid metabolism and lipid accumulation are characteristic hallmarks of renal cell carcinoma (RCC). While there is prior evidence closely linking such lipid accumulation within RCC cells and consequent tumorigenesis, the mechanisms underlying this process remain incompletely understood. In this study, a series of bioinformatics analyses were initially performed by screening RCC databases and gene sets, ultimately leading to the identification of TRIB3 as an oncogene that functions as a central regulator of lipid metabolism. TRIB3 overexpression was observed in both RCC patient tumor tissues and cell lines, and this upregulation was correlated with a worse RCC patient prognosis. When TRIB3 was knocked down, this resulted in a reduction in lipid accumulation and the consequent induction of endoplasmic reticulum (ER) stress-related apoptotic cell death. At the molecular level, interactions between TRIB3 and PLIN2 were found to abrogate TEB4-mediated PLIN2 ubiquitination and consequent degradation, thus maintaining higher PLIN2 expression levels. This simultaneously helps facilitate the accumulation of lipids while preserving ER homeostasis, thus driving accelerated RCC tumor progression. This TRIB3-PLIN2 axis thus represents a promising new target for efforts to treat RCC.

## Introduction

Renal cell carcinoma (RCC) tumors, which arise from renal tubular epithelial cells, are among the most commonly developed urological malignancies, as they comprise 80–90% of all renal cancer diagnoses [1]. The primary pathological subtypes of RCC include clear cell RCC (ccRCC), papillary RCC, and chromophobe RCC, among which ccRCC is the most common such that it accounts for an estimated 70–80% of RCC diagnoses [2]. While surgery can be an effective means of treating localized RCC, this cancer type often exhibits a poor prognosis, particularly in cases where patients are only diagnosed when the disease is already relatively advanced. A range of therapeutic options have been proposed for advanced metastatic RCC patients, including anti-angiogenic tyrosine kinase inhibitors and T-cell checkpoint therapies. Despite these options, however, effectively treating RCC remains challenging owing to substantial variability with respect to targeted drug tolerance and patient survival, together with relatively poor immunotherapy response rates [3]. As such, there remains a pressing need to conduct new mechanistic studies focused on the incidence and progression of RCC so that more effective diagnostic and prognostic biomarkers for this cancer type can be defined, potentially enabling the design of new treatments and combination regimens aimed at improving the outcomes of affected patients.

The accumulation of high levels of lipids is a major hallmark of ccRCC that is the cause of its “clear” appearance, thus serving as the etiological basis for the naming of this tumor type [4]. Lipids primarily accumulate within ccRCC cells in lipid droplets (LDs) consisting of an outer surface protein-containing phospholipid monolayer that encapsulates triglycerides, cholesteryl esters, and other neutral lipids [5]. Despite intensive research interest focused on abnormal lipid metabolism that arises in ccRCC tumors, the significance of these changes and their underlying mechanisms are not adequately understood in the context of ccRCC initiation and progression. Only recently has evidence emerged that aberrant lipid accumulation is essential for the development of ccRCC tumors [6]. In tumor cells, abnormally high lipid concentrations have been found to stabilize the structure of the endoplasmic reticulum (ER), protect against ER stress, and enhance cellular activity so as to facilitate disease progression [7]. As such, lipid metabolism-focused research has emerged as a key topic of interest in the kidney cancer research field, although additional research will be essential to clarify the mechanisms that give rise to the dysregulated lipid metabolism observed within ccRCC cells.

The Tribbles pseudokinase family consists of a series of stress-related proteins that harbor a serine/threonine kinase domain but that are catalytically inactive owing to the absence of an ATP binding site or key aspartate and lysine residues. In humans, this family consists of TRIB1, TRIB2, and TRIB3, which exhibit a high degree of homology and structural similarity [8]. TRIB3 consists of a C-terminal protein-binding domain, a central pseudokinase domain that is highly conserved across species, and an N-terminal region. Although it lacks any intrinsic kinase activity, TRIB3 can nonetheless function as a regulatory or scaffold protein that shapes key processes, including glycolipid metabolism, differentiation, proliferation, and cellular stress responses [9]. Given its status as a cell stress sensor, TRIB3 upregulation is detected under a range of adverse conditions, including nutrient deprivation, hypoxia, and ER stress, wherein it serves as an essential component of the responses that aim to mitigate the damaging effects of these stressors, thus preserving cellular viability [10].

Here, TRIB3 was identified as an oncogene associated with lipid metabolism that is involved in the pathogenesis of RCC, with TRIB3 upregulation corresponding to poor prognostic outcomes. At the mechanistic level, the binding of TRIB3 to PLIN2 was found to disrupt the ability of TEB4 to promote the ubiquitin modification and consequent degradation of PLIN2. The resultant stabilization of PLIN2 and associated LD accumulation helps to maintain appropriate ER homeostasis, thus facilitating RCC progression.

## Materials and methods

### Cell culture

The 786-O, A498, ACHN, and Caki-2 RCC cell lines, as well as control HK-2 cells, were obtained from The American Type Culture Collection (ATCC, USA). These cells were cultured at 37 °C in a 5% CO_2_ incubator in high-glucose DMEM containing 10% FBS and 1% penicillin–streptomycin.

### Tissue samples

A total of 12 paired ccRCC patient tumor and paracancerous tissue samples collected from 2019–2022 were obtained from the Department of Urology of the Seventh Affiliated Hospital of Sun Yat-sen University. None of the selected patients received targeted therapy, immunotherapy, chemotherapy or radiotherapy before their tumors were surgically removed. Samples were snap-frozen with liquid nitrogen upon collection and stored at −80 °C for future analysis. A tissue microarray (TMA) consisting of 90 paired ccRCC patient tumors and adjacent normal tissue samples was purchased from Shanghai Outdo Biotech Company, which also provided information regarding patient clinical staging and pathological diagnosis. The Medical Ethics Committee of the Seventh Affiliated Hospital of Sun Yat-sen University approved this study, and all patients provided informed consent to participate.

### qPCR

TRIzol (Invitrogen) was used to extract total RNA, after which a reverse transcription kit (Takara, Japan) was utilized for cDNA synthesis. QuantiTect SYBR Green PCR master mix (Qiagen) was then utilized based on the provided directions to conduct qPCR analyses, with the 2^−^^ΔΔCT^ method being employed to assess relative gene expression. All primers used for this study are listed in Supplementary Table S1.

### Cellular transfection

The full-length cDNA sequences for human TRIB3, PLIN2, and VHL were amplified via PCR and cloned into the p3XFLAG-CMV-10 vector (Sigma-Aldrich). Short-hairpin RNAs (shRNAs) specific for TRIB3, PLIN2, AIP4, TEB4, and UBR1 were introduced into the pLKO.1-puro vector (Sigma-Aldrich). Lipofectamine 3000 (Invitrogen) was used for transfection based on the provided directions. HEK293T cells were used to produce lentiviral particles that were used to generate stably transduced cell lines. Serial virus-containing supernatant-mediated infections were performed over a 72 h period, and puromycin (Invitrogen) was used to select stable RCC cell lines. Empty vector controls and negative control shRNA (shNC) constructs were employed in appropriate experiments (Supplementary Table S1).

### Proliferation and viability analyses

A CCK-8 kit was used to assess the proliferation of cells at specific time points based on provided instructions. Briefly, cells were seeded in 96-well plates (2 × 10^3^/well), followed by the addition of CCK-8 solution to each well and incubation for 15 h. Absorbance at 450 nm was then assessed with a spectrophotometer. Colony formation assays were conducted by plating cells in 6-well plates (800/well) and incubating them for 14 days, followed by fixation with 4% paraformaldehyde (PFA) and staining with 0.5% crystal violet. EdU uptake was assessed as reported previously [11]. Briefly, cells were added to 96-well plates (1 × 10^3^/well) for 24 h, after which EdU (50 μM) was added, and cells were fixed for 15 min with 4% formaldehyde prior to permeabilization for 20 min using 0.5% Triton X-100. After rinsing with PBS, 100 μL of 1× Apollo reaction cocktail was added, cells were incubated for an additional 30 min, and they were then imaged via fluorescent microscopy (Leica, Germany).

### GST pull-down assay

Glutathione-Sepharose 4B bead (Samgon Biotech, China)-coupled GST fusion proteins and cellular lysates were incubated together for 2 h at 4 °C, followed by bead washing with GST binding buffer. Bound proteins were then separated via SDS-PAGE, with subsequent immunoblotting using appropriate antibodies.

### Immunohistochemistry (IHC)

IHC staining was performed as in prior reports [12]. After fixing tissues using 4% formalin, they were embedded in paraffin, cut into 4 μm sections, treated to eliminate endogenous peroxidase activity and nonspecific protein binding, and incubated overnight at 4 °C with primary antibodies. Sections were then washed with PBS, incubated with HRP-conjugated secondary antibody for 1 h at 37 °C, stained with DAB, and counterstained for 3 min with hematoxylin. Investigators then assessed slides in a blinded manner based on the frequencies of positive cells (0: 0%, 1: 1–25%, 2: 26–50%, 3: 51–75%, 4: 76–100%) and the intensity of staining (0: negative, 1: weak, 2: moderate, 3: strong). These two scores were then multiplied together to calculate the final IHC scores.

### Murine xenograft experiments

BALB/c nude mice (4 weeks old, Sun Yat-sen University Experimental Animal Center) were each subcutaneously injected with 5 × 10^6^ RCC cells. Tumors were measured once per week for the duration of this study. For metastasis experiments, mice instead received an intravenous injection of 1 × 10^6^ RCC cells via the tail vein, with lungs being collected at 4 weeks after injection. All mice were housed in the Sun Yat-sen University Laboratory Animal Center under specific pathogen-free conditions with controlled temperature and humidity levels. The Sun Yat-sen University Experimental Animal Care Commission approved all studies, which were consistent with both institutional guidelines as well as the NC3Rs ARRIVE guidelines.

### Statistical analysis

GraphPad Prism 8.0 was used for all statistical testing. Data were compared between groups with Student’s *t*-tests and one-way ANOVAs, while correlations were assessed based on Pearson coefficient values. Kaplan–Meier curves were employed to assess patient survival, together with the log-rank test. Quantitative data derived from at least three repeat experiments were presented as means ± SD. *P* < 0.05 was selected as the threshold used to define significance (*P < 0.05; **P < 0.01).

Additional methods can be found in Supplementary Data S1.

## Results

### TRIB3 upregulation predicts poor prognostic outcomes in patients with RCC

**Fig. 1:**
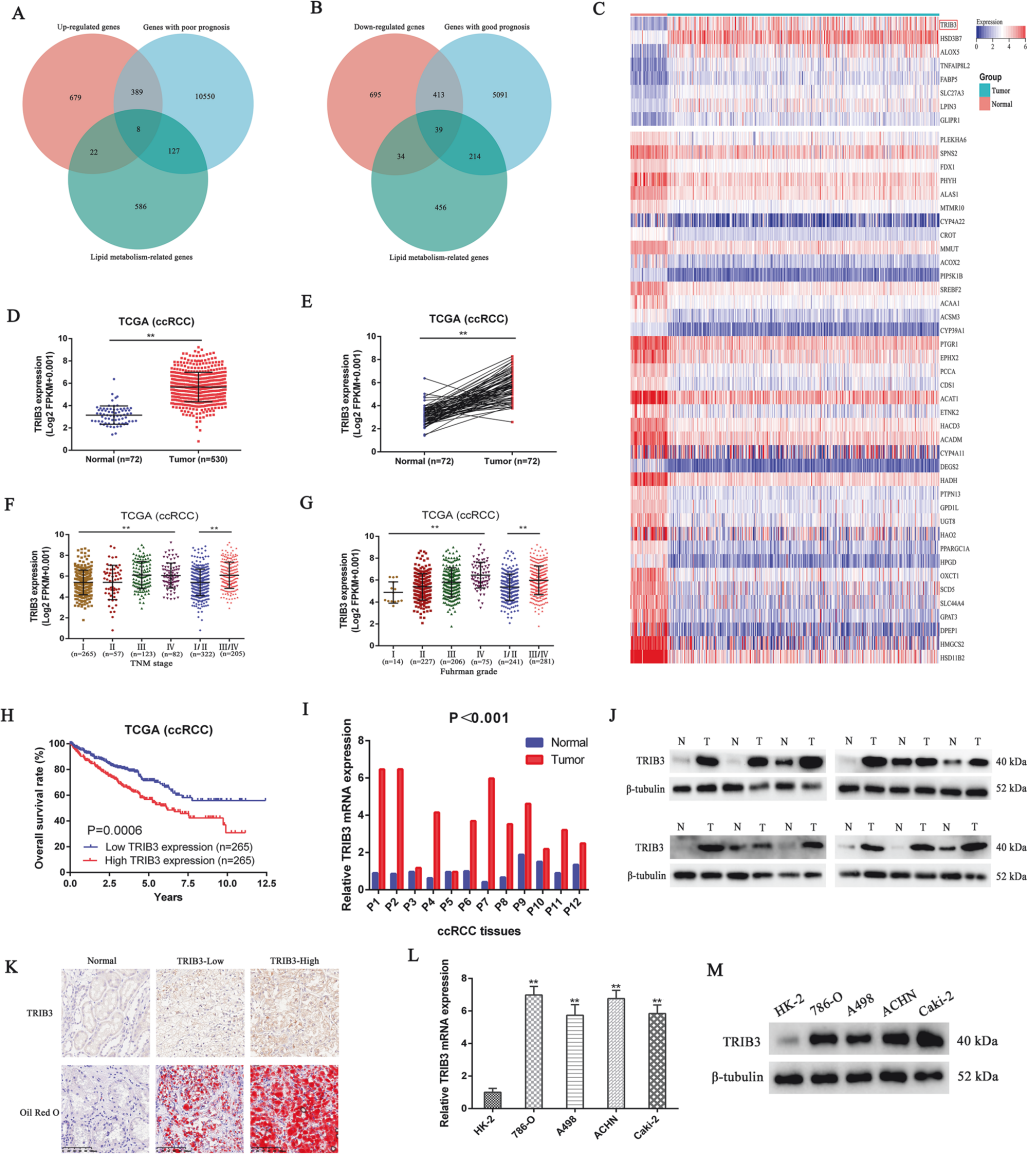
TRIB3 upregulation in RCC predicts poor patient outcomes. **A**, **B** Data from the TCGA database were analyzed to detect major RCC target genes, with a focus on genes exhibiting prognostic value, differential expression, and associations with lipid metabolism in this cancer type. **C** A heatmap representing target genes identified in (**A**, **B**) arranged according to fold-change values for differential expression. **D** TRIB3 mRNA levels were compared between ccRCC tumors (*n* = 530) and normal renal tissue samples (*n* = 70) from the TCGA database. **E** TRIB3 mRNA levels were compared between ccRCC tumors and paired normal tissues (*n* = 70 each) from the TCGA database. **F**, **G** TRIB3 mRNA levels were assessed in patients with specific ccRCC grades and stages using TCGA database data. **H** Correlations between TRIB3 expression and patient overall survival in the TCGA cohort were assessed with Kaplan–Meier curves, using the median TRIB3 expression level to stratify patients into those with low and high TRIB3 expression (*n* = 258 each). **I**, **J** TRIB3 mRNA (**I**), and protein (**J**) levels were assessed in 12 RCC tumor and healthy paracancerous tissue samples. **K** RCC tissues and normal paracancerous tissues were subjected to immunohistochemical staining for TRIB3 and Oil Red O staining. **L**, **M** The mRNA and protein levels of TRIB3 were assessed in five RCC cell lines and one normal cell line. Data are means ± SD. *P < 0.05; **P < 0.01.

To begin assessing the potential importance of lipid metabolism-associated genes in the pathogenesis of RCC, key target genes were initially screened using TCGA data to identify lipid metabolism-associated genes related to RCC patient prognosis that were also differentially expressed in this cancer type. In total, 8 highly expressed putative oncogenes meeting these three criteria were identified (Fig. 1A), as were 39 putative tumor suppressor genes expressed at low levels (Fig. 1B). Of the 8 oncogene candidates, TRIB3 was selected as a target for further study given that it was the most differentially expressed in RCC (Fig. 1C). To confirm this differential expression and to assess the clinical significance of TRIB3 in this oncogenic setting, the TCGA dataset was analyzed to evaluate TRIB3 levels in 530 RCC tumor tissue samples and 72 paracancerous samples. TRIB3 overexpression was detected in this analysis, and this was further confirmed when specifically comparing 72 paired tumor and paracancerous samples from this dataset (Fig. 1D, E). Clinical and prognostic data were additionally analyzed, revealing that higher TRIB3 levels were associated with higher TNM staging and a higher pathological Fuhrman grade among patients with RCC, together with shorter postoperative overall survival (OS) (Fig. 1F–H). TRIB3 was also an independent predictor of RCC patient outcomes in univariate and multivariate Cox regression analyses (Table 1).

**Table 1:** Correlation between the TRIB3 level and the overall survival of ccRCC patients.

Clinical variables	HR	95% CI	*P*-value
*Univariate analysis*			
Age (≥60 vs <60)	1.786	1.304–2.447	0.000
Gender (Male vs Female)	0.951	0.697–1.296	0.749
TNM stage (III/IV vs I/II)	3.875	2.821–5.322	0.000
Furhman grade (III/IV vsI/II)	2.758	1.958–3.884	0.000
TRIB3 (High vs Low)	1.704	1.251–2.320	0.001
*Multivariate analysis*			
Age (≥60 vs <60)	1.532	1.115–2.103	0.008
TNM stage (III/IV vs I/II)	3.015	2.159–4.210	0.000
Furhman grade (III/IV vs I/II)	1.773	1.237–2.540	0.002
TRIB3 (High vs Low)	1.501	1.099–2.049	0.011

To validate these results, TRIB3 mRNA and protein expression levels were assessed in RCC cell lines and tissue samples, revealing its significant upregulation relative to corresponding normal controls in both tissues and cell lines (Fig. 1I-M). Given that TRIB3 is a lipid metabolism-associated gene, lipid accumulation was next assessed via Oil Red O staining. This approach revealed pronounced lipid accumulation in RCC tumor tissues in a manner positively correlated with the expression of TRIB3 (Fig. 1K).

Since the majority of RCC cases exhibit mutations in the von Hippel–Lindau (VHL) tumor suppressor gene, we next evaluated whether TRIB3 upregulation was associated with VHL loss in RCC cells. In order to investigate the impact of VHL on TRIB3, we selected two widely recognized VHL-deficient RCC cell lines, namely 786-O and A498. VHL was overexpressed in these cell lines, and subsequent western blot analysis was conducted to assess alterations in TRIB3 expression. The findings revealed a consistent decline in TRIB3 expression in both 786-O and A498 cells following VHL overexpression (Fig. S1), indicating a potential association between elevated TRIB3 expression and VHL deficiency in RCC cells.

### TRIB3 promotes the aberrant accumulation of lipids and inhibits ER stress-associated death in RCC cells

**Fig. 2:**
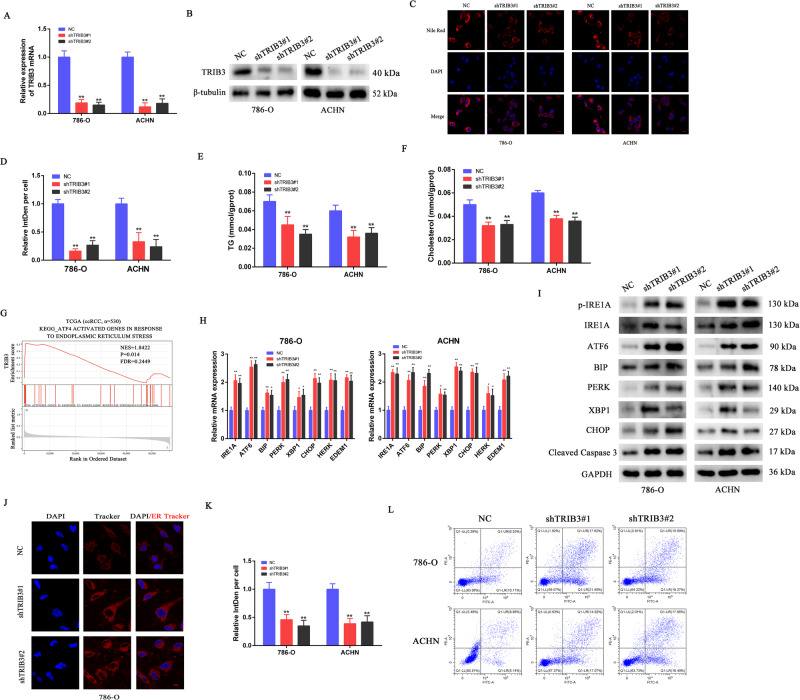
TRIB3 promotes the aberrant accumulation of lipids and inhibits apoptotic death induced by ER stress. **A**, **B** TRIB3 knockdown efficiency in 786-O and ACHN cells was confirmed via Western immunoblotting and qPCR. **C**, **D** Representative fluorescent images and corresponding quantification of Nile Red-stained lipid droplets present in 786-O and ACHN cells with or without the silencing of TRIB3. DAPI was used as a nuclear counterstain. **E**, **F** Lipid accumulation in these TRIB3 knockdown cells was quantified based on cholesterol and triglyceride (TG) measurements. **G** Correlative relationships between ATF4-activated genes in RCC responsive to ER stress and TRIB3 mRNA levels in the TCGA database were assessed through a GSEA approach. Statistical significance was defined by FDR < 0.25 and *P* < 0.05. **H**, **I** Changes in ER stress-related gene expression following TRIB3 knockdown were assessed in 786-O and ACHN cells. **J**, **K** Immunofluorescent analyses were used to assess the ER in TRIB3-knockdown cells; Red: ER-tracker, Blue: DAPI. **L** 786-O and ACHN cell apoptosis following TRIB3 knockdown was assessed via flow cytometry. Data are means ± SD. *P < 0.05; **P < 0.01.

Nile Red staining was further used to assess the effects of TRIB3 on intracellular lipids. Relative mRNA expression levels of TRIB3 were higher in 786-O and ACHN cell lines compared to the other two RCC cell lines (A498 and Caki-2), as indicated by qPCR results (Fig. 1L, M). Therefore, we chose these two cell lines, which exhibited the highest TRIB3 expression, to conduct in vitro and in vivo loss-of-function experiments using shRNA constructs, aiming to better elucidate the biological functions of TRIB3. The efficient knockdown of TRIB3 in these cells was confirmed via qPCR and Western immunoblotting (Fig. 2A, B). Nile Red staining indicated that while abundant LD deposits were present in the parental 786-O and ACHN cells, their accumulation was suppressed following the knockdown of TRIB3 (Fig. 2C, D). TG and total cholesterol levels in these cells were also assessed as another measure of lipid accumulation, revealing that TRIB3 knockdown reduced both TG and cholesterol levels in both tested cell lines (Fig. 2E, F).

Deposits of LDs have previously been reported to suppress the induction of a deleterious ER stress response, thus restoring ER homeostasis and enhancing the survival of malignant cells [13, 14]. To explore the impact of TRIB3 on specific gene cells in RCC samples from the TCGA dataset, a GSEA analysis was next performed. This approach revealed an association between TRIB3, as an oncogene, and the ER stress response signaling pathway (Fig. 2G). Consistently, qPCR and Western immunoblotting revealed increased ER stress marker expression in 786-O and ACHN cells following TRIB3 silencing (Fig. 2H, I). ER tracking analyses additionally confirmed that the ER of RCC cells in which TRIB3 had been knocked down was expanded (Fig. 2J, K), in line with the induction of ER stress. Given that ER stress can induce apoptotic death [15, 16], a flow cytometry analysis was performed, which revealed that TRIB3 knockdown resulted in a greater frequency of apoptotic death for both the 786-O and ACHN cell lines (Fig. 2L). Overall, these results suggested that TRIB3 can promote the abnormal accumulation of lipids while inhibiting ER stress and consequent apoptotic death in RCC tumor cells.

### TRIB3 promotes in vitro RCC cell proliferative activity and aggressive growth

**Fig. 3:**
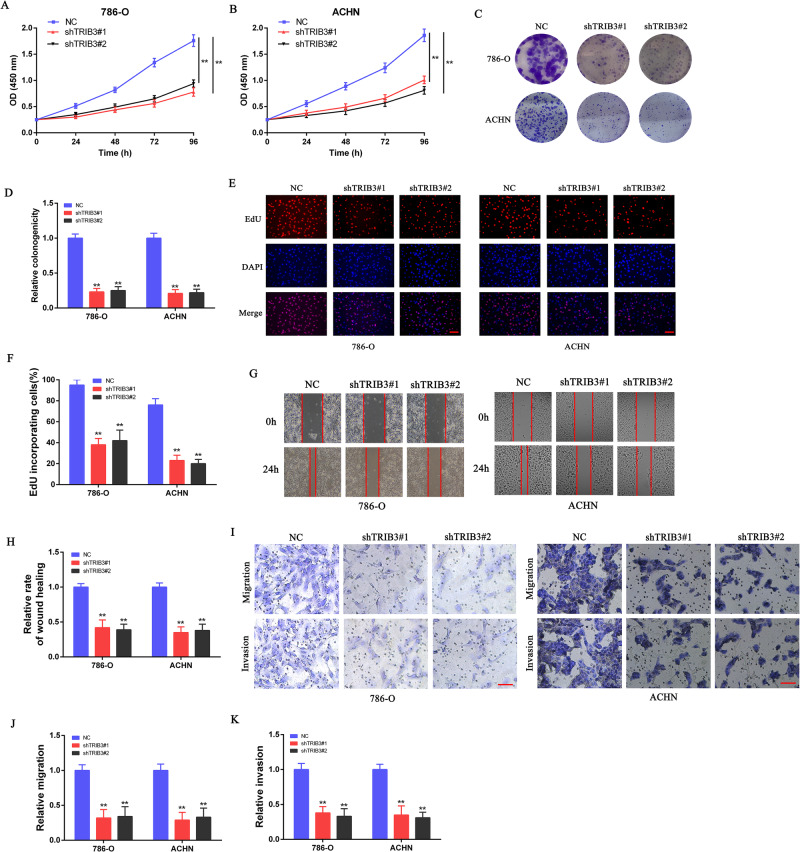
TRIB3 promotes the in vitro proliferative, migratory, and invasive activity of RCC cells. **A**, **B** CCK-8 assays were used to assess 786-O and ACHN cell growth in the presence or absence of TRIB3 silencing. **C**, **D** 786-O and ACHN cells in which TRIB3 was knocked down were evaluated via colony formation assay. **E**, **F** Representative images and corresponding quantification for levels of EdU incorporation into TRIB3-silenced 786-O and ACHN cells. **G**, **H** Wound healing assays were used to analyze the migration of 786-O and ACHN cells following the silencing of TRIB3. **I**-**K** Transwell assays were used to evaluate the migratory and invasive activity of 786-O and ACHN cells following the silencing of TRIB3. Data are means ± SD. *P < 0.05; **P < 0.01.

To examine the functional role played by TRIB3 in RCC, a series of assays, including CCK-8, colony formation, and EdU uptake experiments, were performed. In these analyses, TRIB3 knockdown was associated with the impaired proliferative activity of both 786-O and ACHN cells as compared to shNC-expressing cells (Fig. 3A–F). Wound healing assays additionally revealed the impairment of 786-O and ACHN cellular migratory activity following TRIB3 knockdown (Fig. 3G, H), and both the migratory and invasive activity of these cells was further confirmed to be disrupted by the loss of TRIB3 expression in Transwell-based assays (Fig. 3I–K). Together, these data offer support for the classification of TRIB3 as a pro-tumorigenic mediator that can function in vitro to enhance RCC cell proliferative, migratory, and invasive activity.

### TRIB3 interacts with PLIN2 to promote its stability

**Fig. 4:**
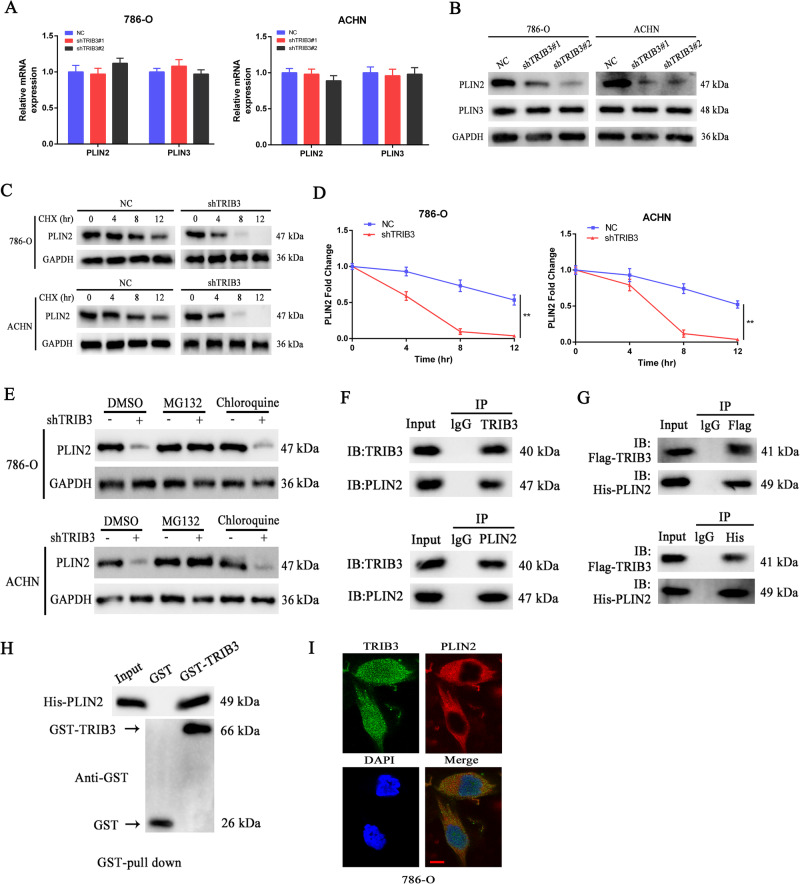
TRIB3 binds to PLIN2 to regulate its protein stability. **A**, **B** qPCR (**A**) and Western immunoblotting (**B**) were used to assess PLIN2 and PLIN3 expression in 786-O and ACHN cells in which TRIB3 was stably knocked down. **C**, **D** Cycloheximide (CHX, 10 μmol/L) was used to suppress protein synthesis in 786-O and ACHN cells exhibiting stable TRIB3 silencing, with time-dependent PLIN2 turnover then being assessed. **E** Proteasome inhibitors (MG132, 20 μM) or lysosome inhibitors (chloroquine, 50 μM) were used to treat 786-O and ACHN cells exhibiting stable TRIB3 silencing, followed by Western immunoblotting-based detection of PLIN2 in these cells. **F** Anti-TRIB3 or anti-PLIN2 were used to immunoprecipitate 786-O cell lysates, and TRIB3 and PLIN2 were then detected via immunoblotting. IP Immunoprecipitation, IB Immnoblot. **G** Flag-TRIB3 and His-PLIN2 expressing HEK293T cells were lysed, and lysates were immunoprecipitated using anti-Flag or anti-His tag, after which Flag-TRIB3 or His-PLIN2 were detected via immunoblotting. **H** His-PLIN2-expressing 786-O cells were lysed, followed by the incubation of these lysates with GST-TRIB3 or GST, after which immunoblotting was used to detect PLIN2-interacting proteins. **I** Immunofluorescent staining was used to assess the localization of PLIN2 and TRIB3 within 786-O cells. Scale bar: 20 μm. Data are means ± SD. *P < 0.05; **P < 0.01.

Members of the perilipin (PLIN) family, which includes PLIN1-5, are the primary protein type associated with LDs. PLIN1 and PLIN4 are the predominant types expressed in mammalian adipose tissue wherein they serve as key regulators of adipocyte lipolysis. PLIN5 expression is primarily evident in oxidative tissues such as the skeletal muscle, whereas PLIN2 and PLIN3, which are responsible for regulating the biogenesis and degradation of LDs, coat these droplets in most other types of cells [17]. As such, PLIN2 and PLIN3 mRNA levels were next assessed in 786-O and ACHN cells, revealing that TRIB3 knockdown had no significant impact on the expression of either of these genes in 786-O or ACHN cells (Fig. 4A). However, TRIB3 silencing was associated with a reduction in the protein levels of PLIN2 in both cell types relative to controls (Fig. 4B). This inconsistency suggested that TRIB3 may function in part by enhancing PLIN2 protein stability. To test this possibility, protein synthesis was inhibited in RCC cell lines in which TRIB3 was stably knocked down via the use of cycloheximide (CHX), enabling the examination of the temporal dynamics of PLIN2 protein levels. In these analyses, TRIB3 knockdown was found to have significantly reduced the half-life of PLIN2 relative to control cells (Fig. 4C, D). This suggests that the TRIB3-mediated enhancement of PLIN2 stability is attributable to the suppression of the degradation of this process. In an effort to clarify the underlying mechanisms governing this enhancement cells in which TRIB3 was knocked down were treated with MG132 or chloroquine to respectively inhibit proteasomal and lysosomal activity. In these experiments, the reductions in PLIN2 levels observed upon TRIB3 knockdown were eliminated by MG132 treatment (Fig. 4E). This is consistent with a model wherein TRIB3 can enhance the protein level stability of TRIB3 via the suppression of ubiquitin/proteasomal pathway-mediated protein degradation.

While TRIB3 lacks any intrinsic kinase activity, it is able to interact with a range of proteins to modulate various signaling processes [18]. To detect potential protein interactions, co-IP assays were next performed using 786-O cells that were or were not transfected with Flag-TRIB3 and His-PLIN2. In these assays, TRIB3 and PLIN2 were found to be complex with one another (Fig. 4F, G). In a glutathione-S-transferase (GST) pull-down assay, the ability of TRIB3 and PLIN2 to directly interact with one another was confirmed (Fig. 4H). Immunofluorescent staining was also used to assess co-localization between TRIB3 and PLIN2, confirming that they co-localize in the cytosol (Fig. 4I). These data provide support for the ability of TRIB3 to directly interact with PLIN2, thereby modulating the ubiquitin/proteasome-mediated degradation of PLIN2.

### TRIB3 disrupts the TEB4-mediated ubiquitination of PLIN2 to stabilize it

Three different E3 ubiquitin ligases have been reported to date as being capable of specifically recognizing PLIN2. In one report, the AIP4 E3 ligase was found to be recruited to LDs by Spartin, whereupon it modulates the ubiquitination status of PLIN2 and a range of other lipid-related proteins [19]. In another study, the knockout of the Drosophila E3 ubiquitin ligase TEB4-encoding CG1317 gene in HeLa cells was found to modulate PLIN2 ubiquitin modification and consequent degradation [20]. A third report demonstrated that amino acid deficiency in hepatocytes can trigger the inactivation of the UBR1 E3 ligase such that it can no longer catalyze PLIN2 polyubiquitination and degradation, resulting in PLIN2 upregulation that interferes with hepatic fat catabolism and gives rise to fatty liver disease [21].

**Fig. 5:**
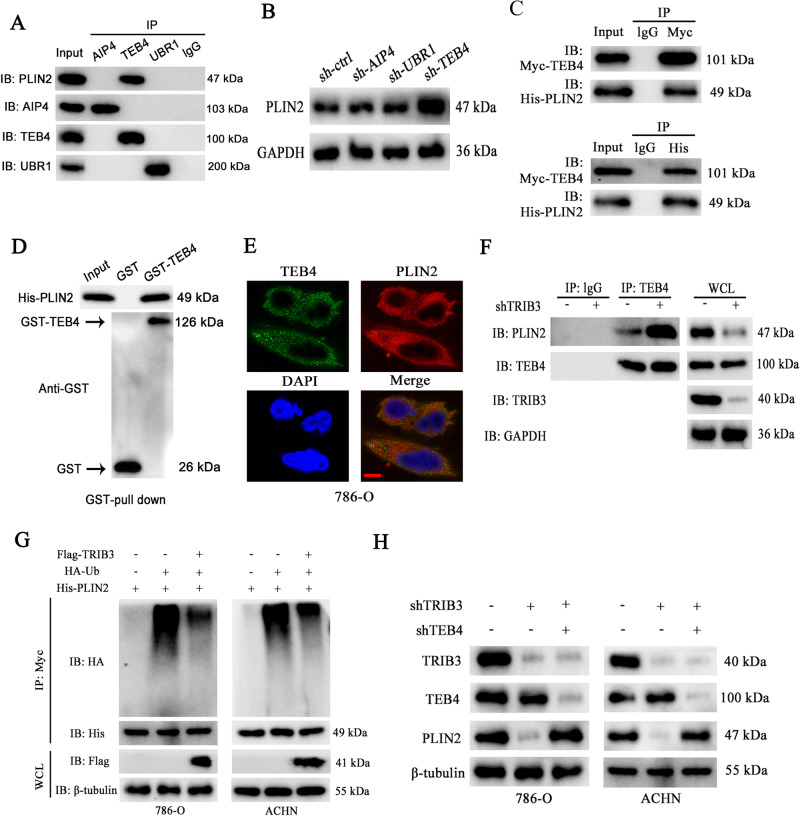
TRIB3 interacts with PLIN2 to disrupt its TEB4-mediated ubiquitination. **A** The ability of endogenous PLIN2 to interact with UBR1, TEB4, and AIP4 was assessed via Co-IP using samples prepared from 786-O cells. **B** PLIN2 levels were detected via Western immunoblotting following AIP4, TEB4, or UBR1 knockdown. **C** Anti-Myc or anti-His were used to immunoprecipitate lysates prepared from 786-O cells expressing Myc-TEB4 and His-PLIN2, after which these tagged proteins were detected via Western immunoblotting. **D** Lysates prepared from 786-O cells expressing His-PLIN2 were incubated with GST-TEB4 or GST, and PLIN2 interactions were then detected via Western immunoblotting. **E** Immunofluorescence was used to examine the localization of PLIN2 and TEB4 within 786-O cells. Scale bar: 20 μm. **F** Co-IP analyses of endogenous PLIN2 and TEB4 in cells in which TRIB3 was or was not silenced. **G** 786-O and ACHN cells were transfected with HA-Ub and His-PLIN2 with or without Flag-TRIB3 to evaluate the ubiquitin modification of TRIB3. Cells were treated with MG132 (10 μM) to inhibit proteasomal activity 6 h before collection. **H** PLIN2 protein levels were detected within 786-O, and ACHN cells transfected with or without shTRIB3 and shTEB4 via Western immunoblotting. Data are means ± SD. *P < 0.05; **P < 0.01.

To test the potential involvement of these three PLIN2-recognizing E3 ligases (AIP4, TEB4, and UBR1) in the ubiquitin-mediated degradation of PLIN2 in RCC cells, a co-IP assay was next performed seeking to detect the ability of PLIN2 to interact with these ligases. These analyses revealed that PLIN2 was only able to interact with TEB4 within RCC cells (Fig. 5A). Consistent with these findings, the shRNA-mediated silencing of TEB4 resulted in an increase in PLIN2 protein levels in these cells, whereas AIP4 or UBR1 silencing had no impacts (Fig. 5B). Co-IP analyses confirmed the ability of TEB4 and PLIN2 to interact in 786-O cells expressing Myc-TEB4 and His-PLIN2, supporting a functional interaction between the two (Fig. 5C). This direct interaction was also validated in a GST pull-down assay (Fig. 5D). Immunostaining revealed the cytosolic co-localization of TEB4 and PLIN2 within 786-O cells (Fig. 5E). As such, these results suggested that TRIB3 is capable of stabilizing PLIN2 through its ability to regulate its TEB4-catalyzed ubiquitin modification. Strikingly, TRIB3 silencing resulted in the enhancement of PLIN2-TEB4 binding without any corresponding change in the protein levels of TEB4 within 786-O cells (Fig. 5F). Following the co-transfection of 786-O and ACHN cells with HA-Ub and His-PLIN2 with or without Flag-TRIB3, a significant reduction in PLIN2 ubiquitination was detected following the upregulation of TRIB3 (Fig. 5G). The simultaneous knockdown of TREB3 was also sufficient to reverse TRIB3 silencing-induced PLIN2 repression (Fig. 5H). These results support a model wherein TRIB3 is able to stabilize PLIN2 in RCC cells by inhibiting its TEB4-catalyzed ubiquitination.

### PLIN2 is essential for the TRIB3-mediated progression of RCC tumors

**Fig. 6:**
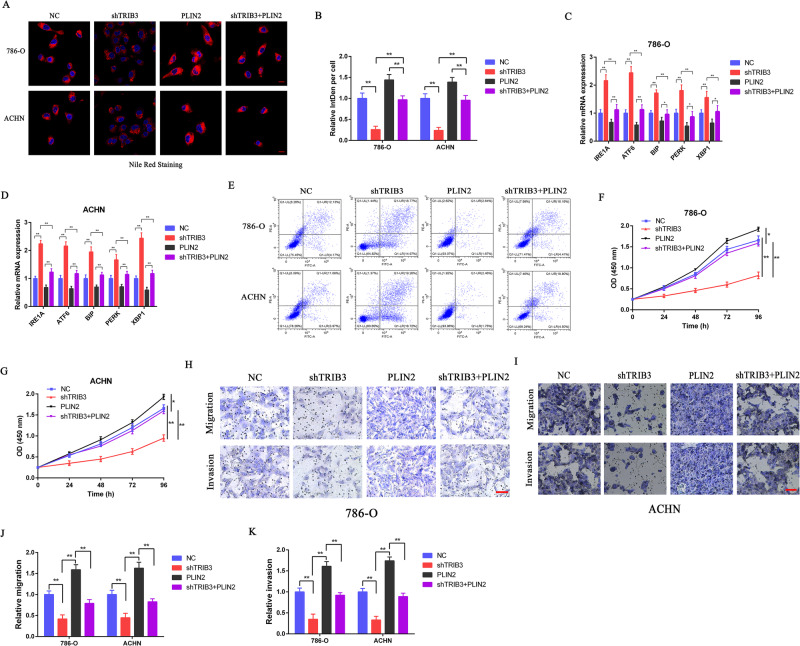
TRIB3 mediates tumor progression in a PLIN2-dependent manner. **A**, **B** Representative fluorescent images and corresponding quantification of Nile Red-stained lipid droplets present in 786-O and ACHN cells with or without the silencing of TRIB3 and the expression of PLIN2. DAPI was used as a nuclear counterstain. **C**, **D** ER stress-related gene expression was assessed in 786-O and ACHN cells following transfection with or without shTRIB3 and PLIN2. **E** The apoptotic death of cells transfected with or without shTRIB3 and PLIN2 was evaluated via flow cytometry. **F**, **G** CCK-8 assays were used to assess the growth of cells transfected with or without shTRIB3 and PLIN2. **H**, **I** Representative images of Transwell migration and invasion assay results using 786-O and ACHN cells transfected with or without shTRIB3 and PLIN2. **J**, **K** Quantification of the data from (**H**, **I**). Data are means ± SD. *P < 0.05; **P < 0.01.

The ability of TRIB3 to suppress the breakdown of PLIN2 suggested the possibility that PLIN2 may be required for the TRIB3-mediated progression of RCC. To test this possibility, PLIN2 was overexpressed in cells in which TRIB3 had been knocked down to conduct a rescue experiment. In these cells, Nile Red staining, qPCR, and flow cytometry revealed that PLIN2 was able to partially reverse TRIB3 knockdown-related apoptosis, ER stress marker upregulation, and reductions in LD deposition (Fig. 6A–E). Consistently, PLIN2 overexpression enhanced 786-O and ACHN cell proliferative, migratory, and invasive activity (Fig. 6F–K). These data thus support the ability of TRIB3 to facilitate the progression of RCC in a manner that is at least partially dependent on PLIN2.

### TRIB3 promotes in vivo RCC progression in a PLIN2-dependent fashion

**Fig. 7:**
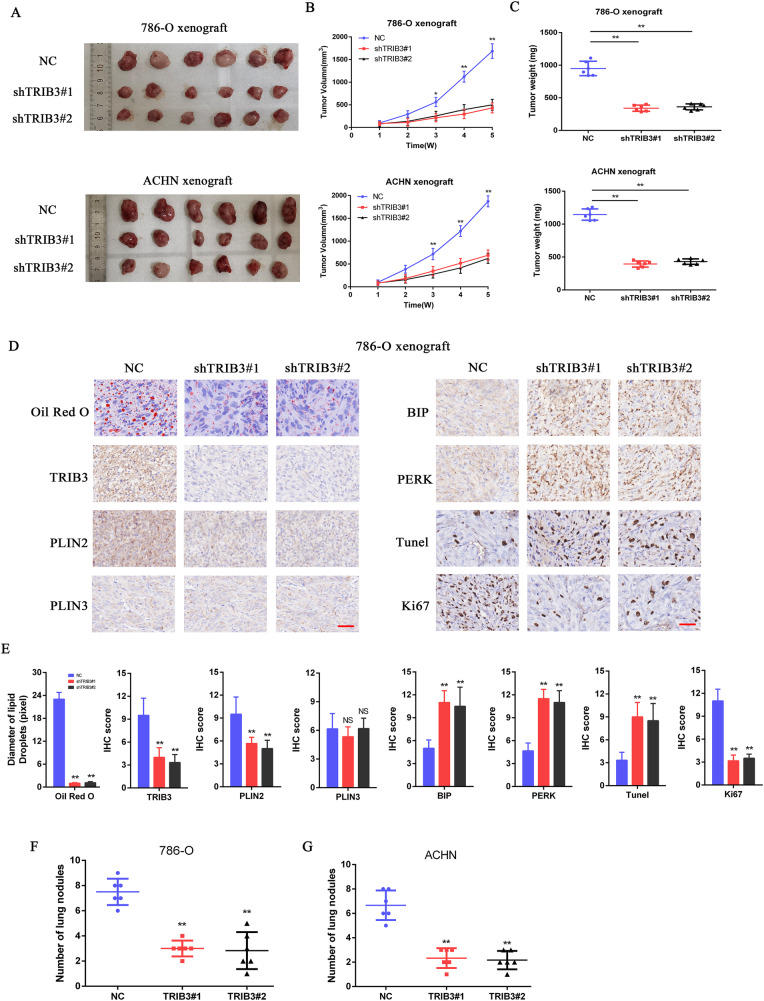
TRIB3 promotes in vivo RCC tumor progression via PLIN2. **A** Gross tumor images after resection from xenograft model mice subcutaneously implanted with 786-O or ACHN cells in which TRIB3 was knocked down. **B** Tumor volume was measured on a weekly basis for 5 weeks in tumor-bearing nude mice. **C** Final tumor weights were assessed after tumor resection. **D** IHC staining was used to assess tumor tissues for proteins related to ER stress, apoptotic death, and proliferative activity. Lipid accumulation was measured via Oil Red O staining. **E** Quantitative analysis of the data shown in (**D**). **F**, **G** Numbers of metastatic lung nodules in the indicated groups. Data are means ± SD. *P < 0.05; **P < 0.01.

To better understand how TRIB3 influences the pathogenesis of RCC in vivo, a xenograft tumor model was next established through the subcutaneous axillary implantation of nude mice with RCC cells in which TRIB3 was or was not silenced. TRIB3 silencing was associated with significant reductions in tumor size and weight relative to control tumors (Fig. 7A–C). The TRIB3-related inhibition of PLIN2 at the protein level was also confirmed in this in vivo model system. IHC staining revealed that PLIN2 levels were reduced in TRIB3-silenced tumors, whereas the same was not true for PLIN3. Ki-67 staining was significantly suppressed following TRIB3 knockdown, whereas pronounced increases in BIP and PERK protein levels were evident in these same tumors, consistent with a role for TRIB3 as a pro-proliferative protein that can suppress ER stress induction. To test the effects of endogenous TRIB3 on in vivo cell survival, TUNEL staining was additionally conducted, revealing that TRIB3 silencing was associated with a greater frequency of apoptotic cells as compared to control conditions. Oil Red O staining additionally revealed that LD deposits in TRIB3-silenced tumors were significantly reduced in abundance, and TG and cholesterol tests additionally revealed that lipid accumulation was decreased in these RCC tumors following TRIB3 knockdown (Fig. 7D, E). The metastatic potential of these tumors was additionally evaluated through a tail vein injection model of metastasis in nude mice. Consistent with in vitro data, TRIB3 silencing suppressed RCC cell metastasis (Fig. 7F). Together, these data support the ability of TRIB3 to facilitate the PLIN2-mediated progression of RCC.

### ccRCC tumors exhibit pronounced TRIB3 and PLIN2 overexpression

**Fig. 8:**
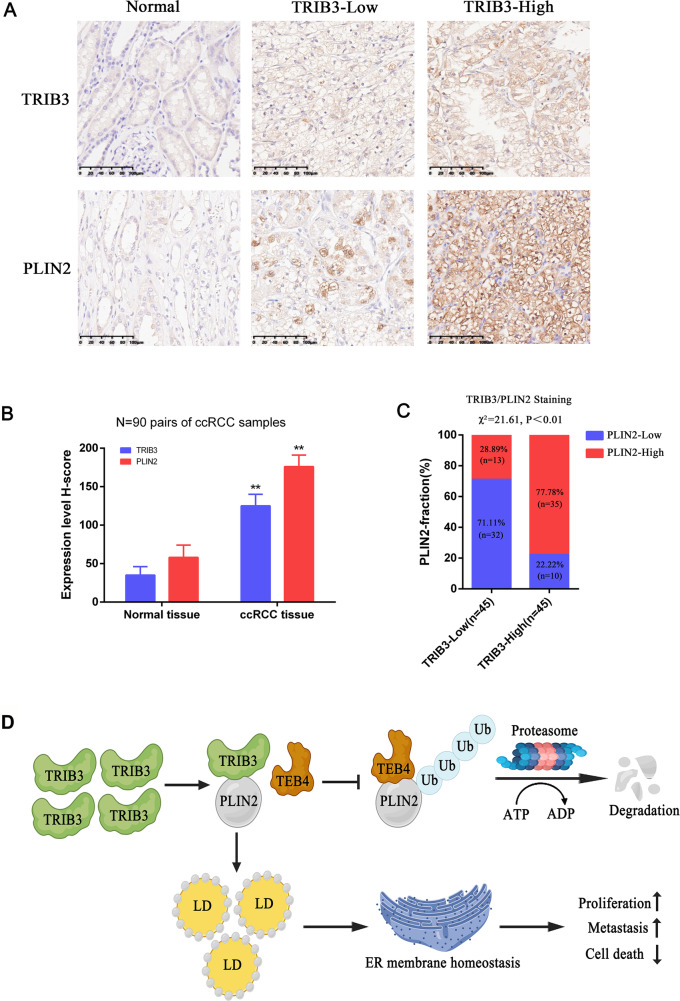
TRIB3 and PLIN2 protein levels are correlated within ccRCC tumors. **A**, **B** Representative IHC images of TRIB3 and PLIN2 (**A**) with corresponding quantitative analyses of *H*-score values for ccRCC tumors and paired healthy tissue from a 90-donor tissue microarray. Members of this ccRCC cohort were grouped based on the median *H*-score value. **C** Correlations between TRIB3 and PLIN2 protein levels were assessed via the *χ*^2^ test. **D** Proposed model illustrating the protective function of TRIB3/PLIN2-mediated LD accumulation in RCC progression. Data are means ± SD. *P < 0.05; **P < 0.01.

To better clarify the clinical relevance of TRIB3 and PLIN2 in ccRCC, a TMA was next leveraged to assess the levels of these proteins in 90 pairs of ccRCC patient tumors and matched normal tissues [22]. TRIB3 expression was confirmed to be significantly increased in RCC tumors relative to matching control samples (Fig. 8A, B). When the staining intensity of ccRCC patients stratified into groups with low or high levels of TRIB3 or PLIN2 expression, as determined based on median H-score values, low TRIB3 staining was confirmed to be associated with weaker PLIN2 staining in these tumors, while higher TRIB3 levels were associated with more intense PLIN2 staining (Fig. 8A–C). In summary, these findings highlight strong clinical correlations between the expression of TRIB3 and PLIN2 in RCC.

## Discussion

ccRCC is a form of renal malignancy that is characterized by persistent dysregulated lipid metabolism and the consequent accumulation of abnormally high levels of lipids [23]. The resultant LDs play key roles in the maintenance of cellular homeostasis, enabling ccRCC cells to remain viable such that disease progression can occur [24]. The most effective means of remediating these abnormal lipid changes, however, has yet to be determined. Here, TRIB3 was identified as a key lipid metabolism-associated gene in initial bioinformatics analyses of ccRCC patient samples. In subsequent mechanistic analyses, TRIB3 was found to promote the accumulation of LDs in tumor cells through its ability to stabilize PLIN2, thereby alleviating ER stress and facilitating more rapid progression of ccRCC. At the molecular level, the ability of TRIB3 to sustain PLIN2 protein levels was found to be the result of its ability to inhibit PLIN2 ubiquitination by the TEB4 E3 ubiquitin ligase.

As a pseudokinase protein, TRIB3 lacks intrinsic kinase activity and does not possess any ATP binding pocket or catalytic core. Despite this fact, TRIB3 engages in a range of protein-protein interactions that have been reported to be biologically important for several signaling pathways [25]. The atypical pseudokinase domain harbored by TRIB3 can function as a binding platform for substrates that can be recognized by E3 ubiquitin ligases in a context-specific manner [26]. By interacting with various components of the intracellular signaling machinery, TRIB3 can reportedly promote oncogenesis and persistent inflammatory activity. For example, by interacting with the autophagic receptor p62 it is capable of interfering with autophagic activity and normal ubiquitin-proteasome system activity in a manner conducive to the development and progression of cancer [27]. TRIB3 can also enhance PML-RARα while regulating its degradation [28]. Here, TRIB3 was found to be capable of interacting directly with PLIN2 and regulating its ubiquitin-mediated proteasomal degradation.

High-fat diets have been linked to the exacerbation of ccRCC onset and progression in prior epidemiological research [29], as these diets provide a rich supply of substrates for adipocytes that can enable the synthesis of LDs and contribute to the incidence of obesity, which necessitates lipid reduction. Tumor cells with dysregulated lipid metabolism must similarly reduce lipid levels through the regulated accumulation of LDs [30]. These droplets primarily consist of neutral fats, including TGs and cholesterol esters, surrounded by a phospholipid bilayer associated with a range of proteins [5], including PLIN family proteins. In prior studies, LD accumulation has been shown to facilitate ER stabilization, thus supporting the survival and proliferation of tumor cells [7]. Here, TRIB3 knockdown within RCC cells was sufficient to significantly abrogate the LD deposits within these cells, resulting in the enhancement of ER stress-related apoptotic death in a manner that ultimately inhibits the ability of tumors to grow and metastasize.

Previous research efforts have shown that the ubiquitin-proteasome system can facilitate PLIN family protein degradation, with three E3 ligases (AIP4, TEB4, and UBR1) having been found to specifically recognize and ubiquitinate PLIN2 [19–21]. Here, PLIN2 was found to only interact with TEB4 within RCC cells, such that TRIB3 is capable of stabilizing PLIN2 via the inhibition of its TEB4-catalyzed ubiquitin modification.

There are some limitations to this study. First, while TRIB3 expression was assessed in a wide range of RCC cell lines, none were found to exhibit low endogenous TRIB3 levels at baseline, thus precluding gain-of-function experiments aimed at accurately recapitulating the true biological effects of TRIB3 on RCC cells. Secondly, owing to limited access to clinical tissue samples, all analyses focused on the expression of TRIB3 in ccRCC tissue samples, and the correlations between the levels of TRIB3 and patient outcomes were assessed based on data from the TCGA database. Future studies will seek to address these limitations in order to expand the scope of this analysis.

In summary, our research reveals that TRIB3 promotes the progression of RCC by upregulating the lipid droplet-associated protein PLIN2, as shown in Fig 8D. This TRIB3-PLIN2 axis thus represents a promising new target for efforts to treat RCC.

### Supplementary information

Supplementary Data S1

Supplementary Figure S1

Supplementary Table S1

Original image of WB

Reproducibility Checklist

Supplementary Figure Legend

## Data Availability

The datasets used and/or analyzed during this study can be accessed from the corresponding author upon reasonable request.
