# Supplementary material for: TRIB3 promotes the progression of renal cell carcinoma by upregulating the lipid droplet-associated protein PLIN2
Source: Cell Death Dis. 2024 Apr 1;15(4):240. doi: 10.1038/s41419-024-06627-4 (PMC10985002; doi:10.1038/s41419-024-06627-4)
Supplement: Supplementary file 1 — Supplementary Data S1 [file 41419_2024_6627_MOESM1_ESM.docx]

**Supplementary Data S1**

**Supplementary materials and methods**

**Bioinformatics analyses**

Gene expression data from ccRCC patient tumor and paracancerous tissue samples were obtained from the TCGA-KIRC database along with corresponding clinical data, as downloaded from cBioPortal (http://www.cbioportal.org). None of the selected patients had received targeted therapy, immunotherapy, chemotherapy, or radiotherapy prior to surgical removal of their tumors in the TCGA dataset. The clinical staging of RCC patients in the TCGA database was performed according to the tumor staging system established by the American Joint Committee on Cancer (AJCC). The commonly used versions of the tumor staging system were the 7th and 8th editions, which were selected based on the specific time point and data version used in the study. Moreover, the Fuhrman grading system was employed for the pathological grading of RCC patients. Survival curve analysis employed the median as a threshold to differentiate high and low expression groups. A lipid metabolism-related gene list (Table S1) was derived from the Molecular Signatures Database (https://www.gsea-msigdb.org/gsea/msigdb/index.jsp). Pathways enriched in this gene set were assessed via a Gene Set Enrichment Analysis (GSEA) approach, with a false discovery rate (FDR) < 0.25 and a P < 0.05 as the threshold for significance.

**Migration and invasion analyses**

Wound healing assays were performed by growing cells to 100% confluence in 6-well plates and then using a sterile 200 μL tip to generate a scratch wound. After rinsing away injured cells with PBS, cells were cultured in 1% FBS-containing media, and a measurement of wound width was taken every 24 h. For Transwell assays of migratory and invasive activity, cells were cultured for 24 h in serum-free media before addition to the upper portion of the Transwell chamber. Cellular invasivity was assessed using inserts that had been coated with Matrigel (1:8). Following incubation for 24 h, cells were fixed with methanol and stained using 0.05% crystal violet. Cells were imaged via microscopy, and cells in randomly selected fields were counted.

**Nile Red staining**

Intracellular LDs were quantified via confocal microscopy when using a Nile Red Staining Kit (ab228553, Abcam). After preparing the room temperature Nile Red Staining Solution as instructed, culture media was replaced with an equal volume of this staining solution and cells were incubated for 20 min at 37℃ in a 5% CO_2_ incubator. After removing the staining solution, DAPI was used for nuclear counterstaining, followed by confocal microscopic imaging.

**ER staining**

Cells were plated in triplicate confocal microscopy Petri dishes, rinsed twice with HBSS containing Ca^2+^ and Mg^2+^, and incubated for 15 min in warm working solution from the ER-Tracker Red Kit (Servicebio). Cells were then rinsed two times using RPMI-1640, fixed for 5 min with 4% PFA, and nuclei were counterstained with DAPI, followed by confocal microscopic imaging.

**Oil Red O staining**

Saturated Oil Red O solution (Beyotime) was diluted at a 6:4 ratio with dH_2_O to prepare a working solution. Frozen sections of tissue in OCT compound were cut to a thickness of 8 μm, fixed for 10 min with 4% PFA, and then used for staining. Similarly, cells in 6-well plates were rinsed two times using PBS, fixed for 10 min with 4% PFA, and used for staining. In both cases, samples were then treated for 30 s with 60% isopropanol, after which they were stained in the dark at room temperature with Oil Red O staining solution for 10 min. Sections of tissue were counterstained for 2 min using hematoxylin. Samples were rinsed two times, imaged, and counted. LD length in 100 cells was assessed in ImageJ in pixels.

**TG and cholesterol analyses**

Serum or cellular TG or cholesterol levels were assessed with a TG assay kit (Bioengineering) and a Total Cholesterol assay kit (Bioengineering) as per the provided directions. Briefly, an ultrasonic instrument was used to lyse tissue or cell samples, while serum was centrifuged (10 min, 12,000 rpm, 4℃). Then, 2.5 μL of the kit reagent and 250 μL of each sample were combined in 96-well plates and incubated in the dark for 5 min at 37℃, after which absorbance at 510 nm was assessed with a spectrophotometer. A BCA kit was used to detect protein concentrations. All analyses were performed in triplicate.

**TUNEL staining**

A colorimetric TUNEL apoptosis assay kit (Beyotime) was used as instructed to evaluate apoptotic cell death. After staining samples in the TUNEL reaction mixture, they were rinsed and color development was performed with DAB. Six random fields were assessed to count the number of TUNEL-positive cells, with apoptotic indices for each field being computed as the percentage of TUNEL-positive cells to total cells.

**Apoptosis analyses**

A FITC Annexin V Apoptosis Detection Kit (Beyotime) was utilized as instructed to quantify apoptotic cell death with a flow cytometer (FACSCalibur).

**Western immunoblotting**

RIPA buffer with protease inhibitors was used to prepare cell lysates, which were separated via 10-12.5% SDS-PAGE and transferred onto 0.45 μM PVDF membranes. Blots were probed with the following primary antibodies (all from abcam and diluted 1:1000 unless otherwise indicated): TRIB3 (1:10000, ab75846), PLIN2 (1:5000, ab108323), PLIN3 (ab47638), β-tubulin (ab6046), GAPDH (1:5000, ab8245), IRE1 alpha (p-Ser724) (ab308387), IRE1 alpha (3294S, CST), ATF6 (ab83504), BIP (ab21685), PERK (ab229912), XBP1 (ab220783), CHOP (5554S, CST), Cleaved Caspase 3 (1:500, ab32042), TEB4 (PA5-103816, ThermoFisher), AIP4 (ab108515), UBR1 (1:5000, ab108215), VHL (1:1000, ab270968), lgG (ab109489), Flag tag (ab205606), Myc tag (1:800, ab32), His tag (1:5000, ab18184), and HA tag (ab236632). HRP-linked secondary antibodies (1:5000, ab288151/ab97040, abcam) were then used, followed by imaging with ECL-plus Western Blot Detection Reagents.

**Co-immunoprecipitation (Co-IP)**

NP-40 lysis buffer with protease inhibitors was used to lyse cells, after which these lysates were pre-cleared for 2 h with rabbit IgG and immunoprecipitated overnight with appropriate antibodies at 4℃. Protein A/G PLUS-Agarose beads (Santa Cruz) were then added and incubated for 2 h at 4℃, followed by three additional washes with lysis buffer and SDS-PAGE separation. Immunoblotting was then performed as above.

**Immunofluorescent imaging**

RCC cells were cultured on coverslips, fixed for 20 min with 4% PFA, permeabilized for 15 min with 0.1% Triton X-100, rinsed twice with PBS, blocked for 30 min at 37℃ using 5% BSA, and probed overnight with primary antibodies at 4℃. Cells were then rinsed using PBS and incubated for 1 h at 37℃ with appropriate secondary antibodies. DAPI-containing parafilm was then used to seal cells, which were subjected to fluorescent imaging with a confocal microscope.
