# Supplementary figures and images for: TRIB3 promotes the progression of renal cell carcinoma by upregulating the lipid droplet-associated protein PLIN2
Source: Cell Death Dis. 2024 Apr 1;15(4):240. doi: 10.1038/s41419-024-06627-4 (PMC10985002; doi:10.1038/s41419-024-06627-4)

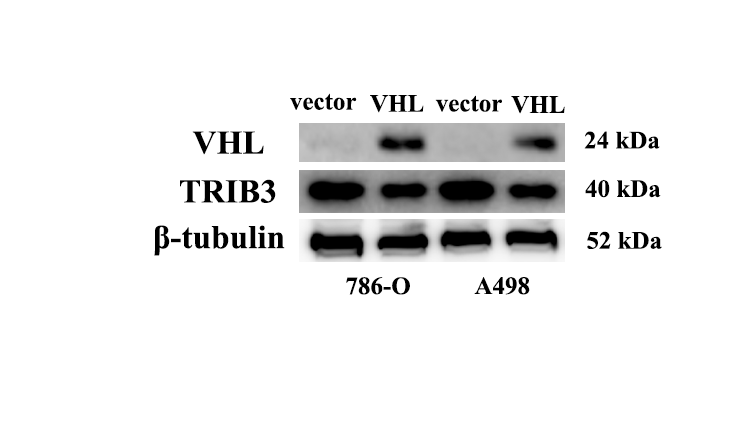

Supplement: Supplementary file 2 — Supplementary Figure S1 [file 41419_2024_6627_MOESM2_ESM.tif]
