# Supplementary material for: TRIB3 promotes the progression of renal cell carcinoma by upregulating the lipid droplet-associated protein PLIN2
Source: Cell Death Dis. 2024 Apr 1;15(4):240. doi: 10.1038/s41419-024-06627-4 (PMC10985002; doi:10.1038/s41419-024-06627-4)
Supplement: Supplementary file 3 — Supplementary Table S1 [file 41419_2024_6627_MOESM3_ESM.docx]

**Supplementary Table S1. The sequences used in this study are listed as follows.**

| **Gene** | **Sequence (5’-3’)** |
| --- | --- |
| **Primers for qRT-PCR** | |
| TRIB3 Forward | TCAAGCTGTGTCGCTTTGTC |
| TRIB3 Reverse | TGTCCCACAGGGAATCATCT |
| PLIN2 Forward | ATGGCATCCGTTGCAGTTGAT |
| PLIN2 Reverse | GGACATGAGGTCATACGTGGAG |
| PLIN3 Forward | TATGCCTCCACCAAGGAGAG |
| PLIN3 Reverse | ATTCGCTGGCTGATGCAATCT |
| IRE1A Forward | CACAGTGACGCTTCCTGAAAC |
| IRE1A Reverse | GCCATCATTAGGATCTGGGAGA |
| AFT6 Forward | TCCTCGGTCAGTGGACTCTTA |
| AFT6 Reverse | CTTGGGCTGAATTGAAGGTTTTG |
| BIP Forward | GAAAGAAGGTTACCCATGCAGT |
| BIP Reverse | CAGGCCATAAGCAATAGCAGC |
| PERK Forward | GGAAACGAGAGCCGGATTTATT |
| PERK Reverse | ACTATGTCCATTATGGCAGCTTC |
| XBP1 Forward | CCCTCCAGAACATCTCCCCAT |
| XBP1 Reverse | ACATGACTGGGTCCAAGTTGT |
| CHOP Forward | GGAAACAGAGTGGTCATTCCC |
| CHOP Reverse | CTGCTTGAGCCGTTCATTCTC |
| HERK Forward | ATGGAGTCCGAGACCGAAC |
| HERK Reverse | TTGGTGATCCAACAACAGCTT |
| EDEM1 Forward | GCTACGACAACTACATGGCTC |
| EDEM1 Reverse | GACTTGGACGGTGGAATCTTT |
| GAPDH Forward | GTGGACATCCGCAAAGACC |
| GAPDH Reverse | CCTAGAAGCATTTGCGGTG |
| **ShRNA oligo sequences** | |
| shTRIB3#1 | CCGGGGTTGGAGTTGGATGACAACTCTCGAGAGTTGTCATCCAACTCCAACCTTTTTG |
| shTRIB3#2 | CCGGGCCCTACAGGCACTGAGTATACTCGAGTATACTCAGTGCCTGTAGGGCTTTTTG |
| sh-PLIN2 | CCGGGTCCTCTCAGGGCAAGATGTTCTCGAGAACATCTTGCCCTGAGAGGACTTTTTG |
| sh-AIP4 | CCGGGGATCACAACTTGGTTCAATGCTCGAGCATTGAACCAAGTTGTGATCCTTTTTG |
| sh-FGFR1 | CCGGGCACACTGTGTGCATTCATCACTCGAGTGATGAATGCACACAGTGTGCTTTTTG |
| sh-UBR1 | CCGGGCAGTAATATGTGACCTAAAGCTCGAGCTTTAGGTCACATATTACTGCTTTTTG |
| scramble | CCGGCAACAAGATGAAGAGCACCAACTCGAGTTGGTGCTCTTCATCTTGTTGTTTTTG |
