# Supplementary material for: TRIB3 promotes the progression of renal cell carcinoma by upregulating the lipid droplet-associated protein PLIN2
Source: Cell Death Dis. 2024 Apr 1;15(4):240. doi: 10.1038/s41419-024-06627-4 (PMC10985002; doi:10.1038/s41419-024-06627-4)

**Original image for Figure 1J**


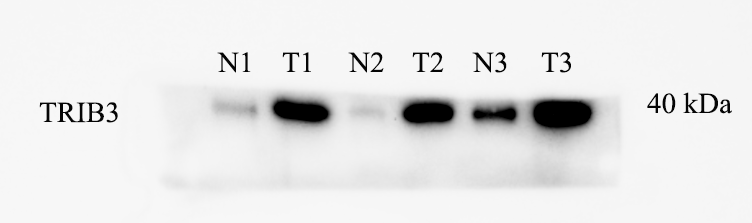


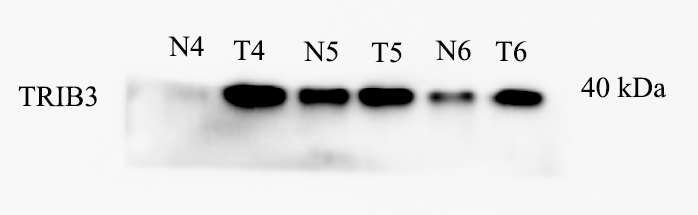


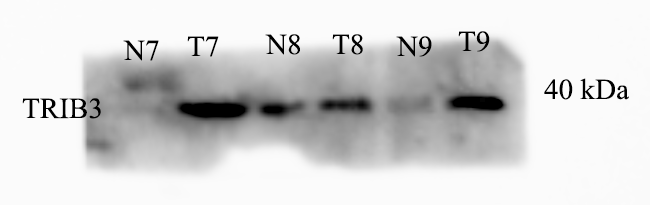


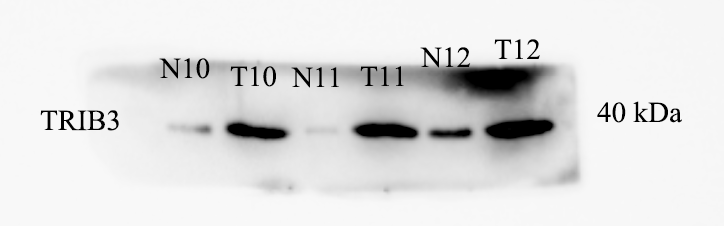


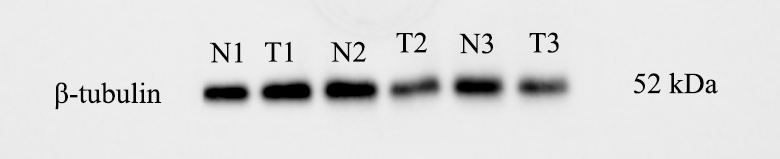


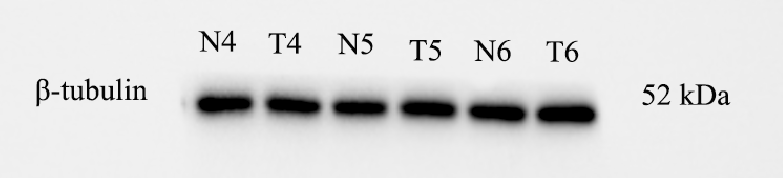


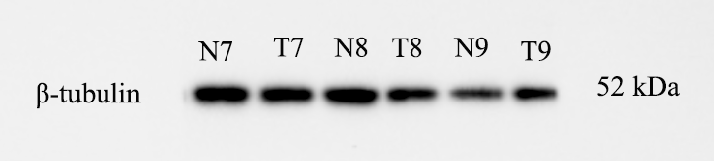


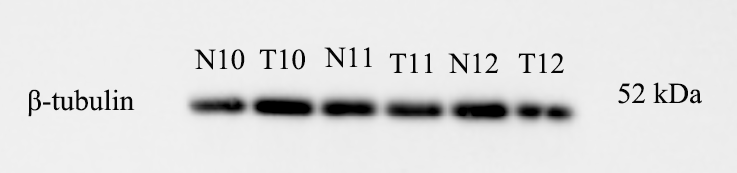


**Original image for Figure 1M**


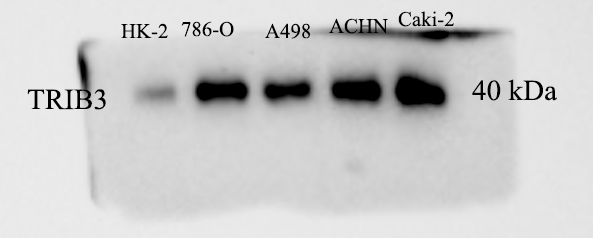


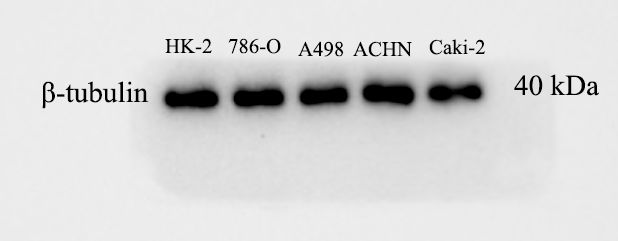


**Original image for Figure 2B**


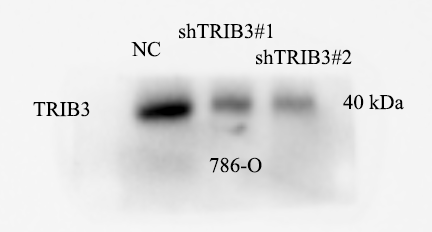


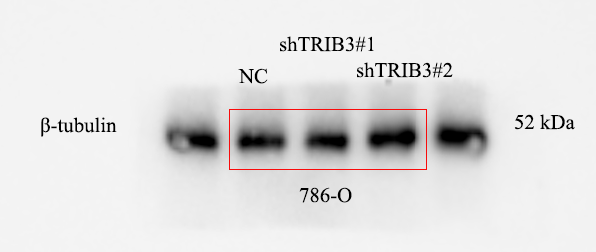


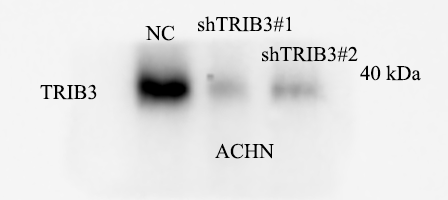


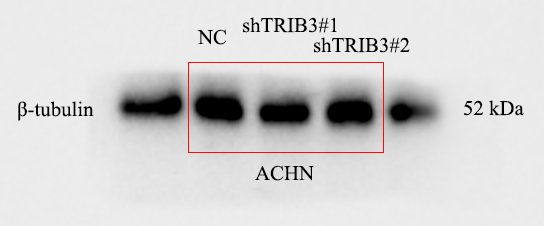


**Original image for Figure 2I**


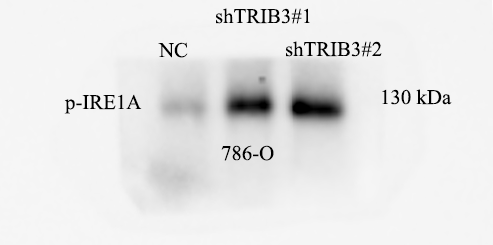


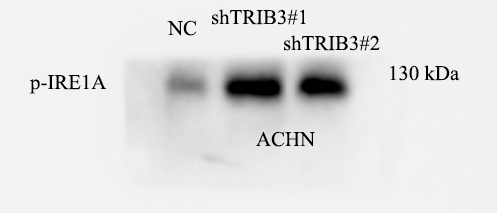


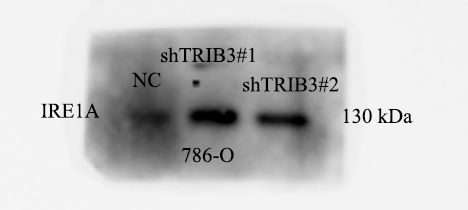


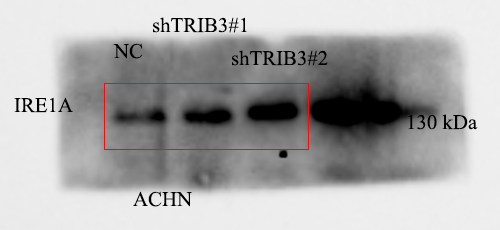


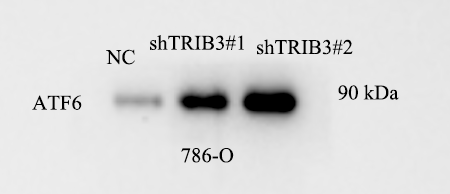


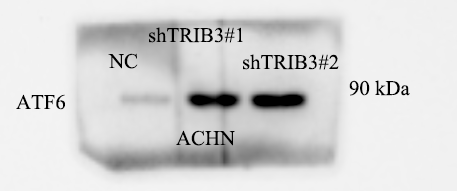


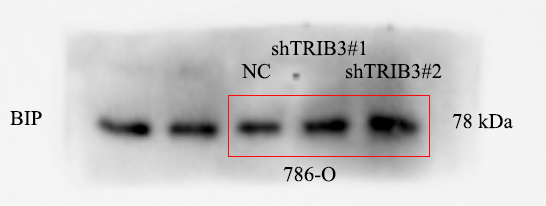


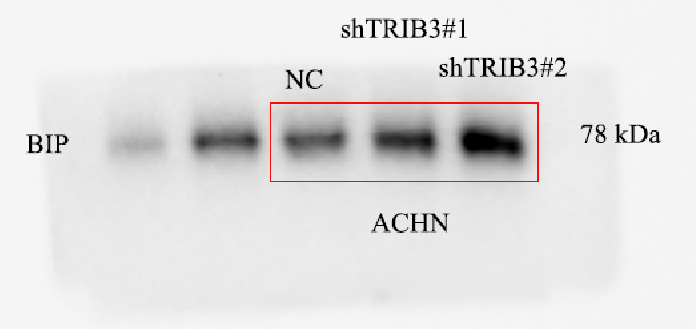


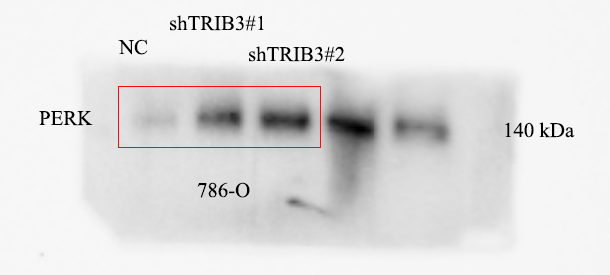


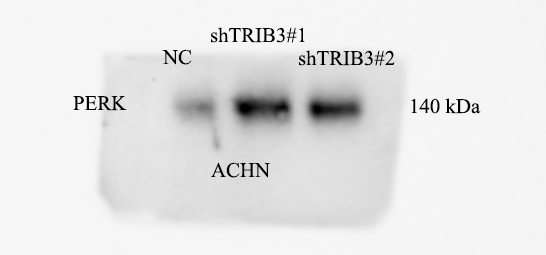


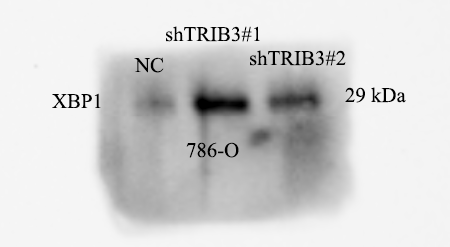


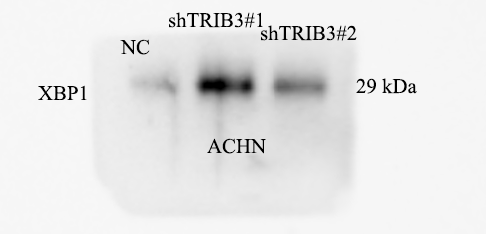


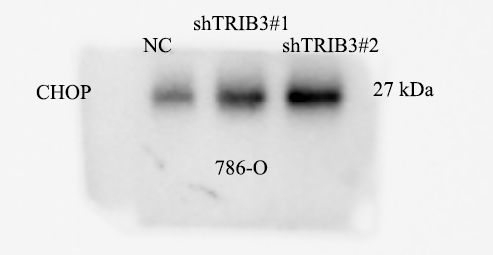


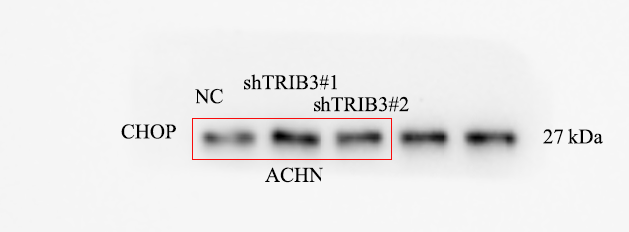


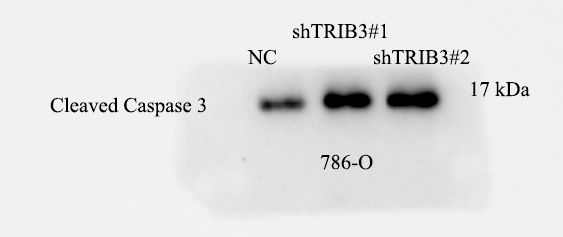


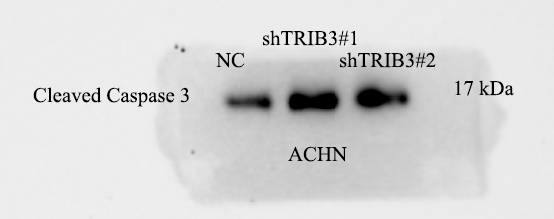


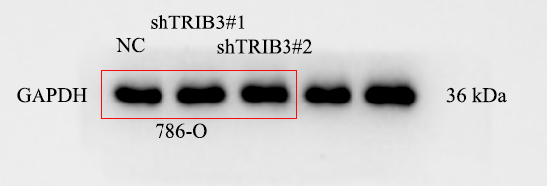


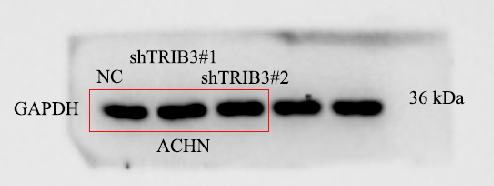


**Original image for Figure 4B**


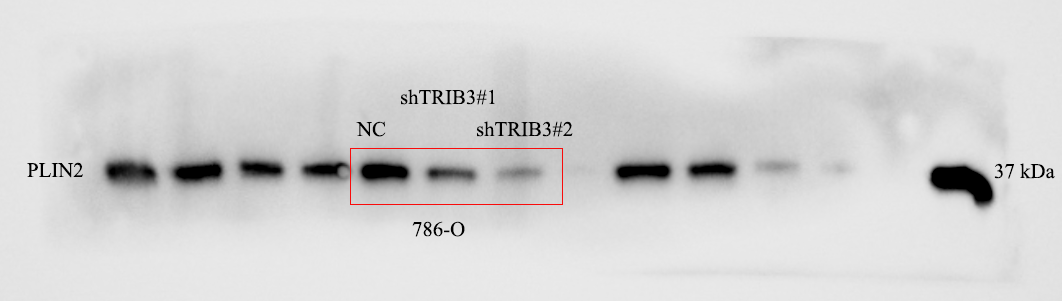


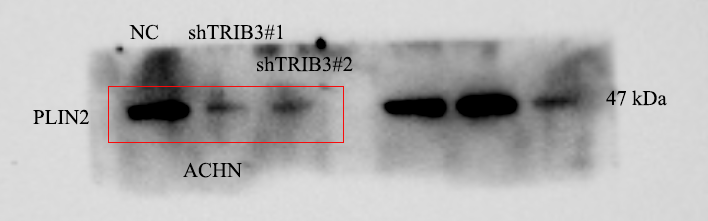


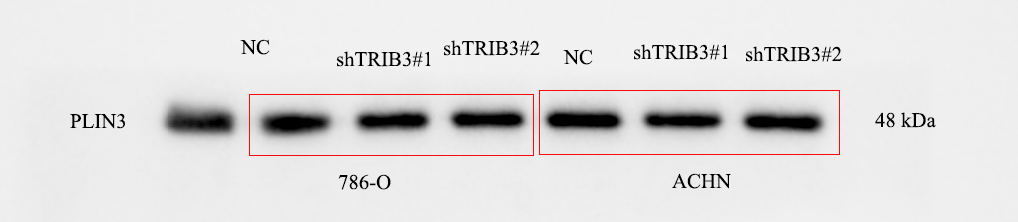


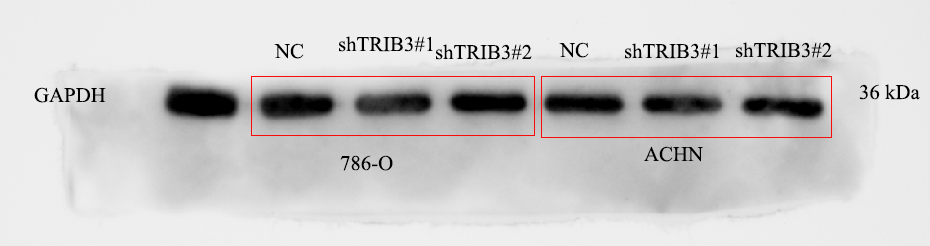


**Original image for Figure 4C**


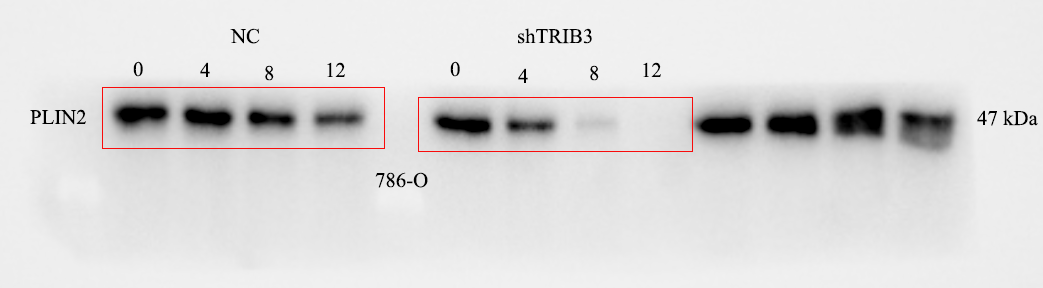


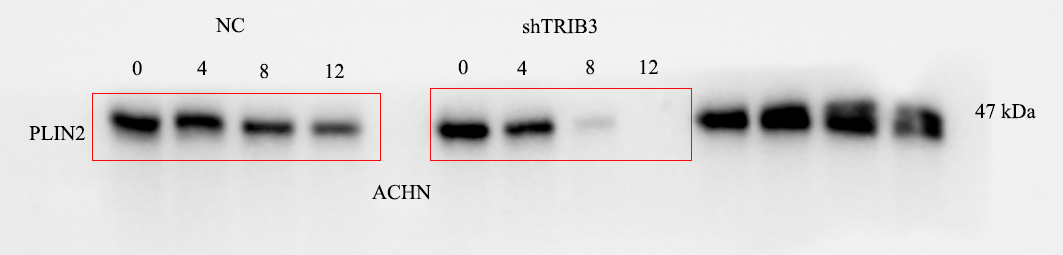


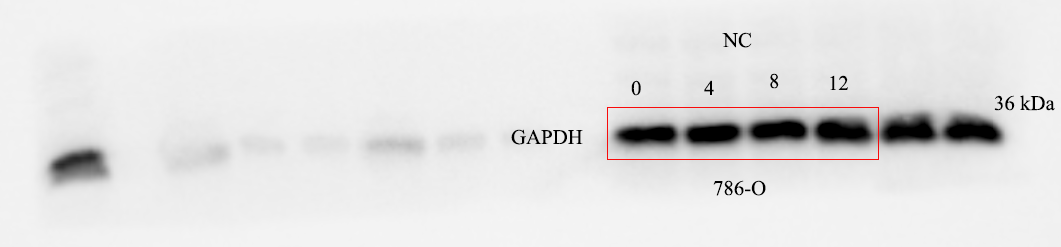


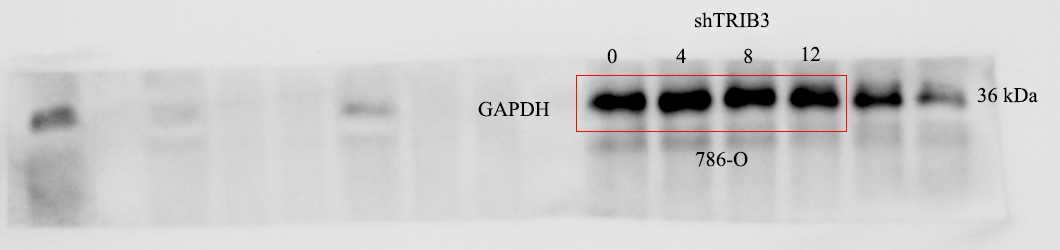


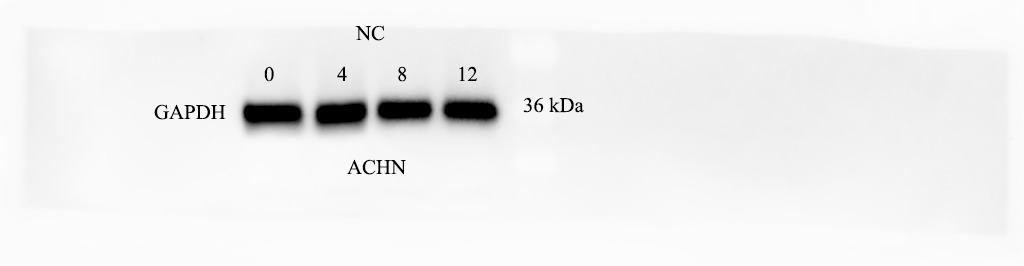


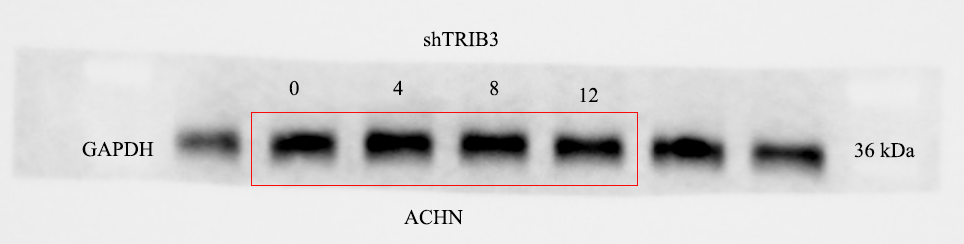


**Original image for Figure 4E**


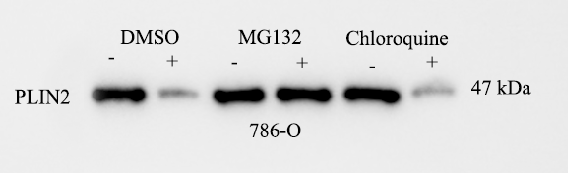


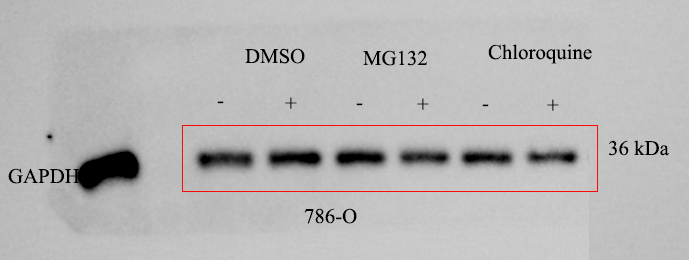


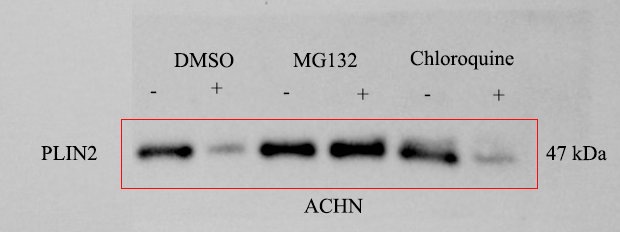


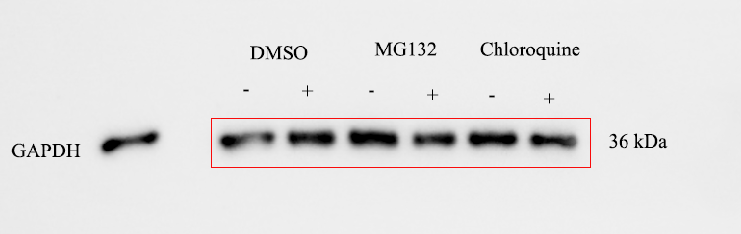


**Original image for Figure 4F**


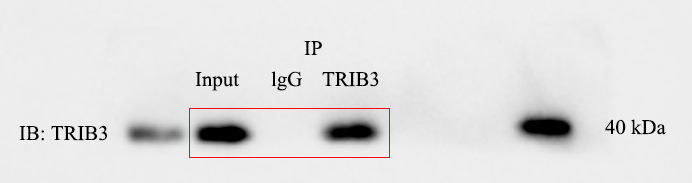


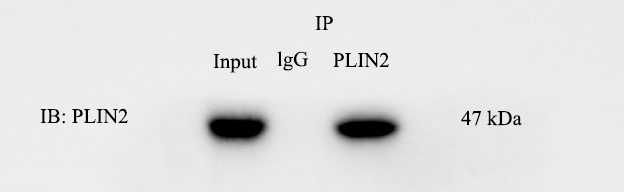


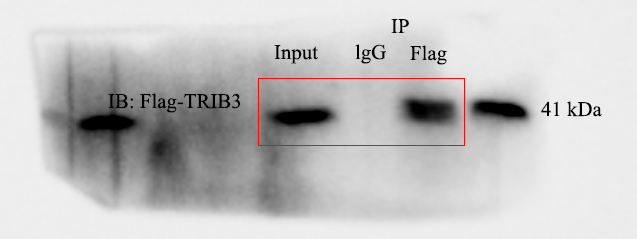


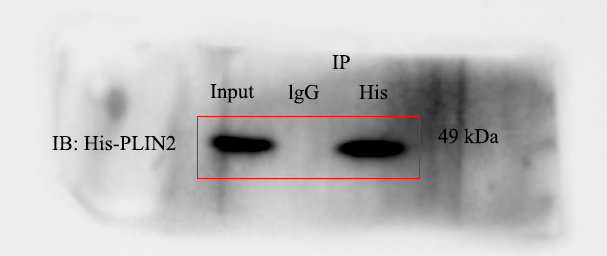


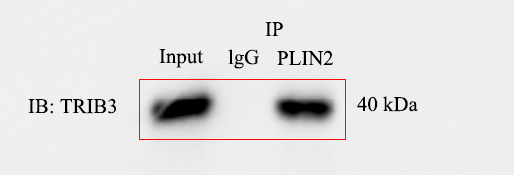


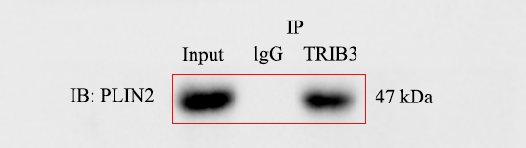


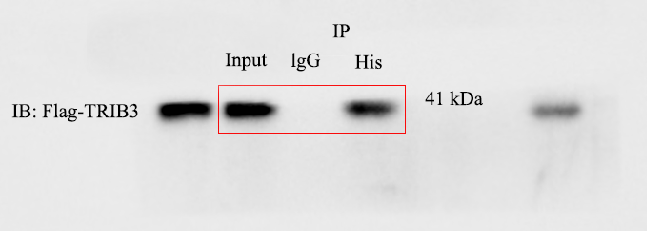


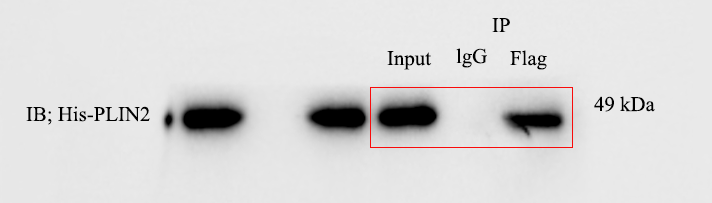


**Original image for Figure 4H**


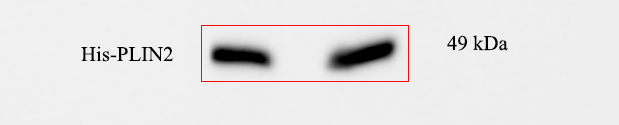


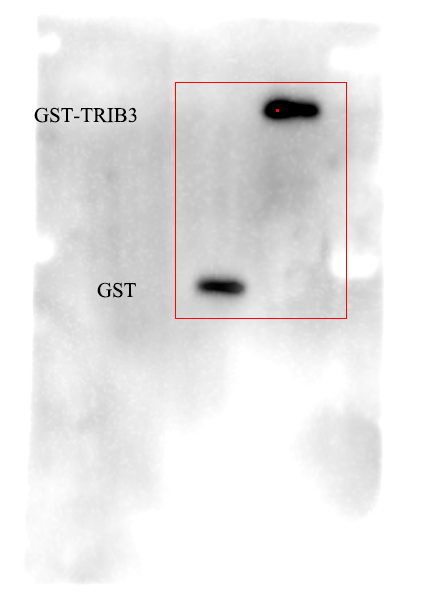


**Original image for Figure 5A**


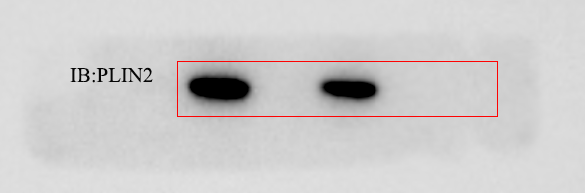


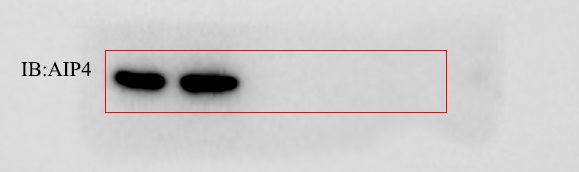


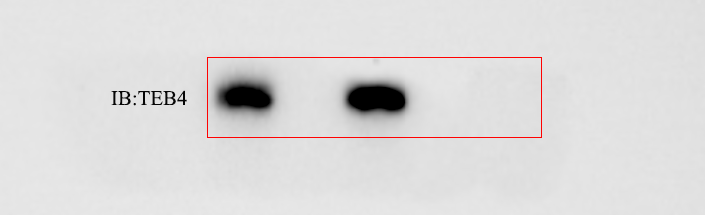


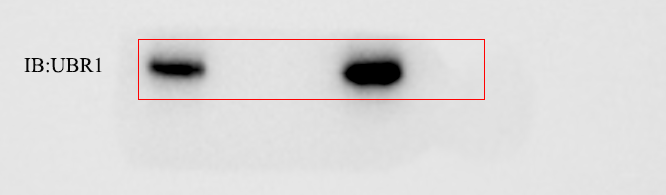


**Original image for Figure 5B**


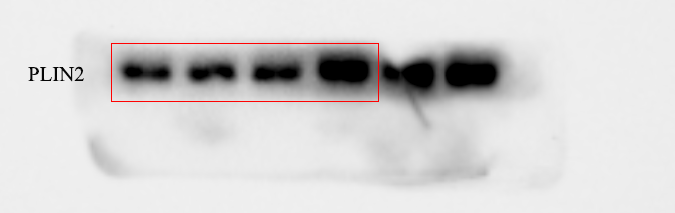


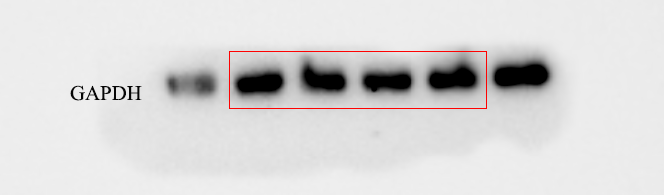


**Original image for Figure 5C**


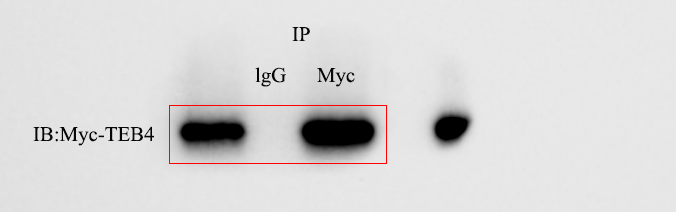


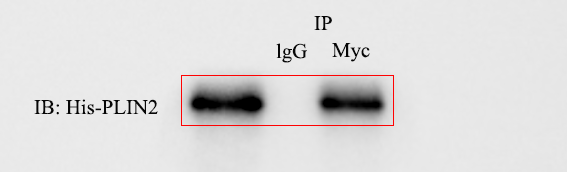


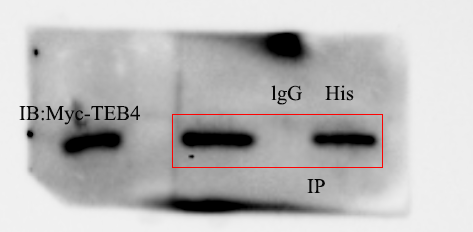


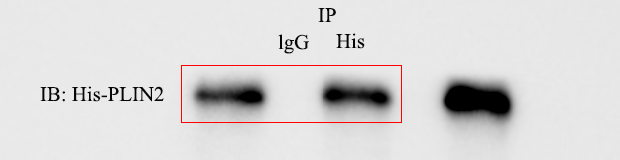


**Original image for Figure 5D**


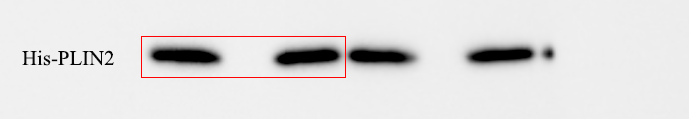


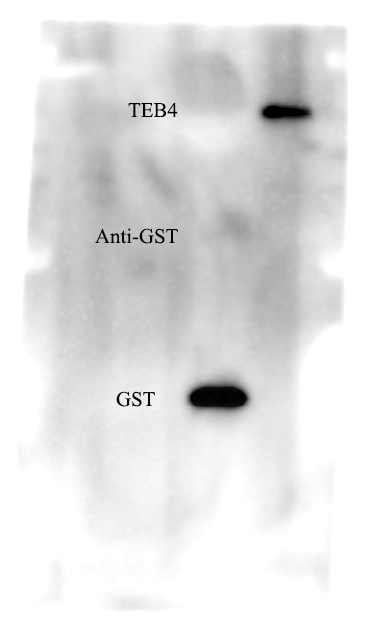


**Original image for Figure 5F**


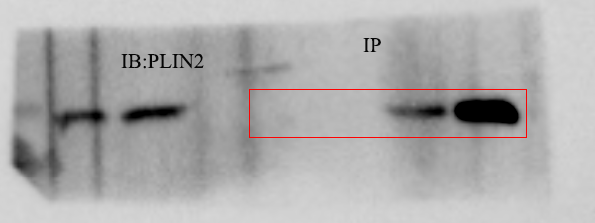


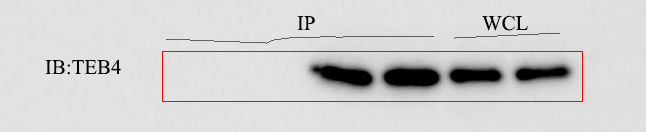


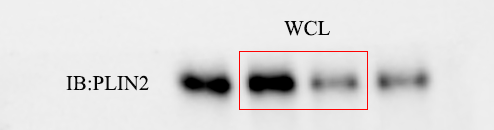


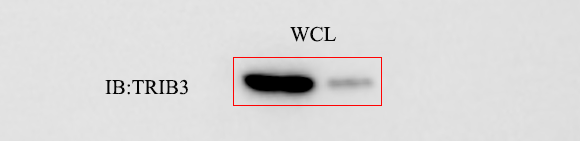


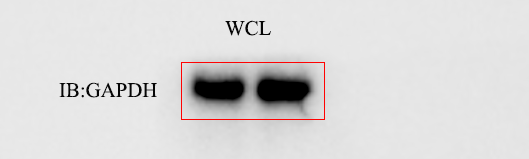


**Original image for Figure 5G**


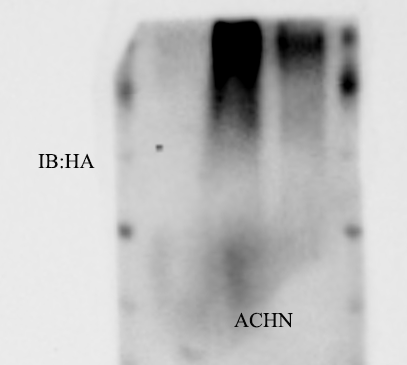

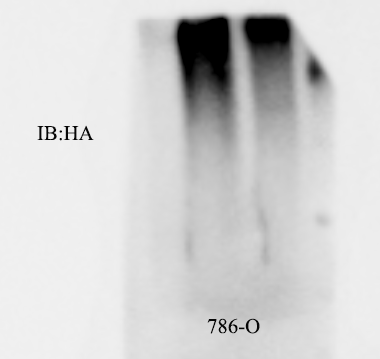


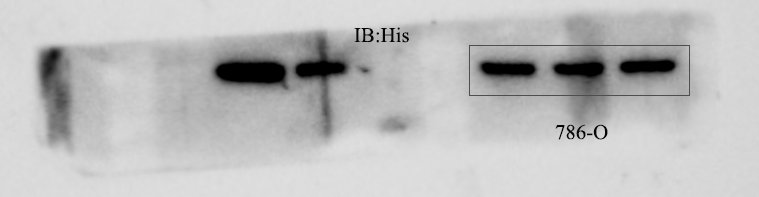


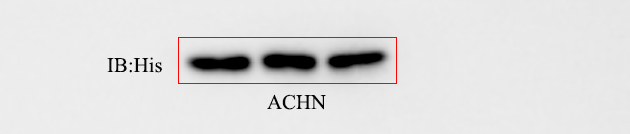


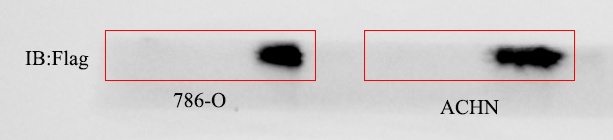


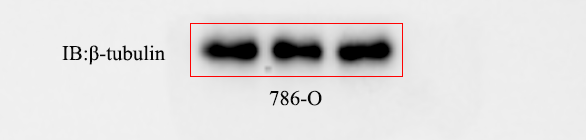


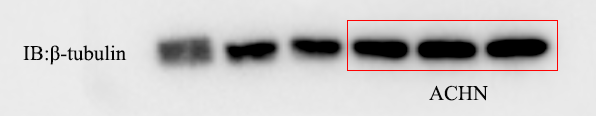


**Original image for Figure 5H**


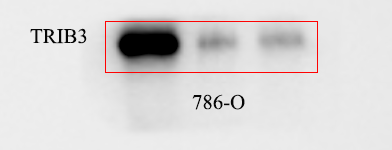


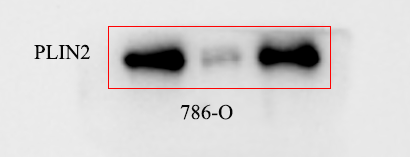


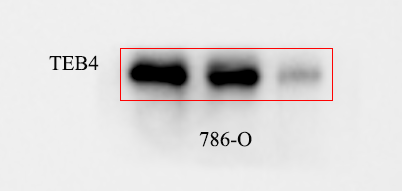


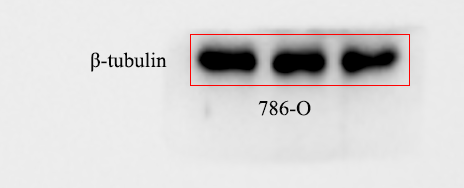


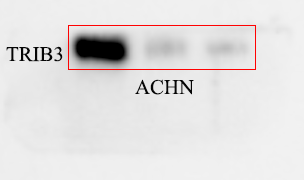


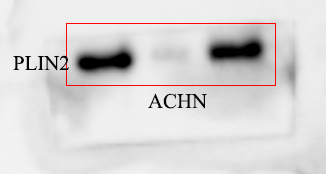


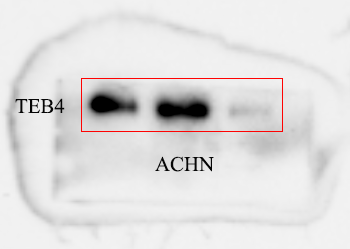


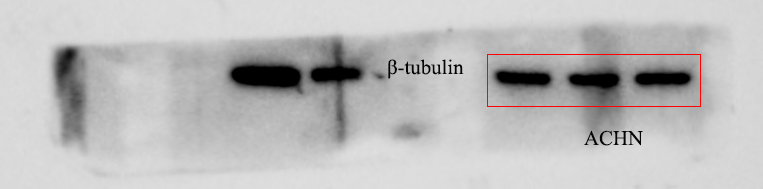


**Original image for Supplementary Figure 1 (Figure S1)**


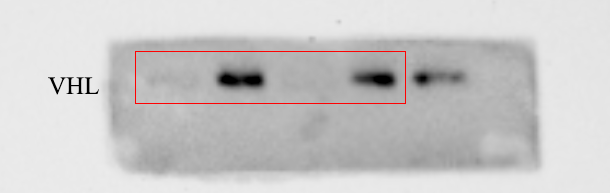


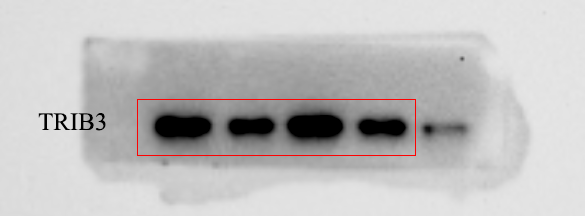


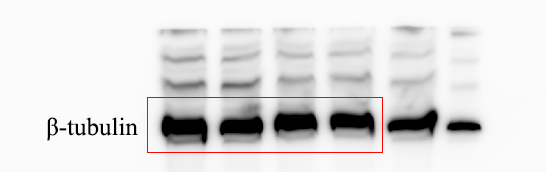

Supplement: Supplementary file 4 — Original image of WB [file 41419_2024_6627_MOESM4_ESM.docx]
