# Supplementary material for: TRIB3 promotes the progression of renal cell carcinoma by upregulating the lipid droplet-associated protein PLIN2
Source: Cell Death Dis. 2024 Apr 1;15(4):240. doi: 10.1038/s41419-024-06627-4 (PMC10985002; doi:10.1038/s41419-024-06627-4)
Supplement: Supplementary file 6 — Supplementary Figure Legend [file 41419_2024_6627_MOESM6_ESM.docx]

Fig S1. Western immunoblotting analysis was conducted to assess TRIB3 expression in VHL-deficient RCC cell lines (786-O and A498) transfected with either an empty vector or VHL.
